# Meta-analysis of epigenome-wide associations between DNA methylation at birth and childhood cognitive skills

**DOI:** 10.1038/s41380-022-01441-w

**Published:** 2022-02-10

**Authors:** Doretta Caramaschi, Alexander Neumann, Andres Cardenas, Gwen Tindula, Silvia Alemany, Lea Zillich, Giancarlo Pesce, Jari M. T. Lahti, Alexandra Havdahl, Rosa Mulder, Janine F. Felix, Henning Tiemeier, Lea Sirignano, Josef Frank, Stephanie H. Witt, Marcella Rietschel, Michael Deuschle, Karen Huen, Brenda Eskenazi, Tabea Sarah Send, Muriel Ferrer, Maria Gilles, Maria de Agostini, Nour Baïz, Sheryl L. Rifas-Shiman, Tuomas Kvist, Darina Czamara, Samuli T. Tuominen, Caroline L. Relton, Dheeraj Rai, Stephanie J. London, Katri Räikkönen, Nina Holland, Isabella Annesi-Maesano, Fabian Streit, Marie-France Hivert, Emily Oken, Jordi Sunyer, Charlotte A. M. Cecil, Gemma Sharp

**Affiliations:** 1 Medical Research Council Integrative Epidemiology Unit (MRC IEU), Bristol Medical School, Population Health Science, University of Bristol, Bristol, UK; 2 Department of Psychology, College of Life and Environmental Sciences, University of Exeter, Exeter, UK; 3 Department of Child and Adolescent Psychiatry/Psychology, Erasmus University Medical Center Rotterdam, Rotterdam, The Netherlands; 4 Lady Davis Institute for Medical Research, Jewish General Hospital, Montreal, QC Canada; 5 Division of Environmental Health Sciences, School of Public Health, University of California, Berkeley, Berkeley, CA USA; 6 Children’s Environmental Health Laboratory, Division of Environmental Health Sciences, School of Public Health, University of California, Berkeley, CA USA; 7 ISGlobal, Barcelona Institute for Global Health, Barcelona, Spain; 8 Universitat Pompeu Fabra (UPF), Barcelona, Spain; 9 CIBER Epidemiology and Public Health (CIBERESP), Madrid, Spain; 10 Department of Genetic Epidemiology in Psychiatry, Central Institute of Mental Health, Medical Faculty Mannheim, University of Heidelberg, Heidelberg, Germany; 11 Epidemiology of Allergic and Respiratory Diseases Team (EPAR), Institute Pierre Louis of Epidemiology and Public Health, UMR-S 1136 INSERM and Sorbonne Université, Paris, France; 12 Department of Psychology and Logopedics, Faculty of Medicine, University of Helsinki, Helsinki, Finland; 13 Department of Mental Disorders, Norwegian Institute of Public Health and Nic Waals Institute of Lovisenberg Diaconal Hospital, Oslo, Norway; 14 The Generation R Study Group, Erasmus MC, University Medical Center Rotterdam, Rotterdam, The Netherlands; 15 Department of Pediatrics, Erasmus MC, University Medical Center Rotterdam, Rotterdam, The Netherlands; 16 Department of Psychiatry and Psychotherapy, Central Institute of Mental Health, Medical Faculty Mannheim, University of Heidelberg, Mannheim, Germany; 17 Center for Environmental Research and Children’s Health (CERCH), School of Public Health, University of California, Berkeley, CA USA; 18 Inserm, Centre for Research in Epidemiology and StatisticS (CRESS), Research Team on Early Life Origins of Health (EAROH), Villejuif, France; 19 Division of Chronic Disease Research Across the Lifecourse, Department of Population Medicine, Harvard Medical School and Harvard Pilgrim Health Care Institute, Boston, MA USA; 20 Department of Translational Research in Psychiatry, Max-Planck-Institute of Psychiatry, Munich, Germany; 21 National Institute of Environmental Health Sciences, National Institutes of Health, Department of Health and Human Services, Research Triangle Park, NC USA; 22 Hospital del Mar Medical Research Institute (IMIM), Barcelona, Spain; 23 Department of Epidemiology, Erasmus MC, University Medical Center Rotterdam, Rotterdam, The Netherlands; 24 Molecular Epidemiology, Department of Biomedical Data Sciences, Leiden University Medical Center, Leiden, The Netherlands

**Keywords:** Genetics, Psychology

## Abstract

Cognitive skills are a strong predictor of a wide range of later life outcomes. Genetic and epigenetic associations across the genome explain some of the variation in general cognitive abilities in the general population and it is plausible that epigenetic associations might arise from prenatal environmental exposures and/or genetic variation early in life. We investigated the association between cord blood DNA methylation at birth and cognitive skills assessed in children from eight pregnancy cohorts within the Pregnancy And Childhood Epigenetics (PACE) Consortium across overall (total *N* = 2196), verbal (total *N* = 2206) and non-verbal cognitive scores (total *N* = 3300). The associations at single CpG sites were weak for all of the cognitive domains investigated. One region near *DUSP22* on chromosome 6 was associated with non-verbal cognition in a model adjusted for maternal IQ. We conclude that there is little evidence to support the idea that variation in cord blood DNA methylation at single CpG sites is associated with cognitive skills and further studies are needed to confirm the association at *DUSP22*.

## Introduction

The human brain starts developing prenatally in the third gestational week and its maturation extends postnatally into late adolescence and most likely adulthood. These processes occur as the result of genetic and environmental factors including the interplay between them [1]. General cognitive ability, or intelligence, often measured as intelligent quotient (IQ), shows considerable heritability, with estimates as high as 20% in infancy, increasing to more than 70% in adulthood [2]. Socioeconomic factors are also associated with cognitive skills and can moderate genetic influences so that their effects vary across socioeconomic strata [3, 4]. It has been shown that cognitive skills predict important long-term outcomes such as higher educational attainment, later mortality, and better physical and mental health [5]. Already in childhood, cognitive functioning is strongly associated with higher educational attainment and later adulthood mortality [6, 7].

Since there is considerable environmental influence on cognitive ability in early childhood [1], it is plausible that environmental exposures that occur in early life, either prenatally or in early childhood, play a role in shaping children’s cognitive development. Early life environmental exposures are known to be revealed in the epigenome, as reflected in changes in DNA methylation marks on cytosine nucleotides followed by guanine (CpG) across the genome. The total heritability of DNA methylation levels from single nucleotide polymorphisms has been estimated to be on average only 19% across the genome, suggesting a strong environmental component [8]. Moreover robust evidence indicate that maternal smoking and folate levels during pregnancy are associated with changes in the child’s epigenome at birth [9, 10]. DNA methylation at birth can therefore reflect exposure to adverse factors, which in turn could have neurodevelopmental consequences.

A recent epigenome-wide association study (EWAS) of cognitive measures in older-aged adults across several cohorts, with final sample sizes ranging between 2557 and 6809 participants, identified associations of DNA methylation with global cognitive function at one intergenic CpG site on chromosome 12 and with phonemic verbal fluency at one CpG site on chromosome 10 in the *INPP5A* gene [11]. Another study of educational attainment in 10767 adults, although not specific to cognitive skills, revealed associations at nine CpG sites which are all known to be associated with smoking [12]. Methylation at each CpG explained 0.3–0.7% of the variance in educational attainment. At present, it is not known whether there is a prospective association between DNA methylation at birth and later cognitive functioning, and whether any association found might be indicative of prenatal exposures rather than environmental exposures across the lifetime such as own smoking habits. Other neurodevelopmental traits have been investigated in relation to DNA methylation at birth. Associations with attention-deficit hyperactivity disorder have been found at five CpG sites in cord blood in a robust meta-analysis [13] and at three sites for social communication trajectories, although the latter were cohort-specific [14]. There was less evidence of associations of DNA methylation at birth with autism spectrum disorder, although there was a strong association with genetic liability for autism [15, 16].

In this study, we extended the previous research on adulthood cognitive function to early life and we aimed to investigate whether DNA methylation was associated with cognitive skills already at birth. As we used a genome-wide approach that interrogates more than 400,000 CpG sites across the genome, we examined associations both independently at each CpG site and at CpG clusters, based on the knowledge that nearby CpG sites are often correlated and that methylation differences might be found across regions spanning multiple CpGs. Specifically we investigated whether: (1) DNA methylation at the single CpG site level in cord blood is prospectively associated with cognitive skills in childhood; (2) DNA methylation at the regional level in cord blood is associated with cognitive skills in childhood.

To assess the biological relevance of our findings and considering that DNA methylation is often tissue-specific and that we did not have access to brain tissue, we also performed a look-up of published brain data to examine the correspondence of DNA methylation between blood and brain at the relevant CpG sites and the potential consequences of DNA methylation differences at these sites on gene expression.

## Methods

### Study sample

The data used in this study were previously obtained from the participants of eight longitudinal birth cohorts within the Pregnancy and Childhood Epigenetics (PACE) Consortium [17] who agreed to contribute to the meta-analysis. The final sample size is therefore determined opportunistically by the number of participants that had available data for this project in each cohort. The cohorts were: Avon Longitudinal Study of Parents and Children (ALSPAC) [18, 19], Center for the Health Assessment of Mothers and Children of Salinas (CHAMACOS) [20, 21], Etude des Déterminants pré et post natals du développement et de la santé de l′Enfant (EDEN) [22], Generation R [23], Infancia y Medio Ambiente project (INMA) [24], Prediction and Prevention of Preeclampsia and Intrauterine Growth Restriction study (PREDO) [25], Pre- Peri- and Postnatal Stress: Epigenetic impact on Depression (POSEIDON) [26], and Project Viva [27]. Participants were all singleton births. Ethical approval for each study was obtained by local committees and consent to use their data was obtained for all participants. Approved researchers with access to individual-level data for each cohort performed in-house analyses and shared only result files with the main analysts. Access to individual-level data is available only upon request to each cohort separately and following local procedures. For more information on each cohort, ethical approval and data access procedures please refer to the Supplementary Material.

### Epigenetic data

For each cohort, cord blood was collected during delivery and DNA was isolated according to standard protocols. DNA was then bisulfite-treated according to standard protocols and loaded onto Infinium HumanMethylation450 BeadChip or Infinium MethylationEPIC arrays (Illumina, San Diego, CA). Array images were scanned and raw methylation intensities were normalized and subjected to quality control according to cohort-specific procedures. For some cohorts, batch correction was applied before analysis (PREDO, INMA and Project Viva), while for the others batch variables were included as covariates in the analysis. The epigenetic data were already pre-processed within each cohort at the time of the analysis, so it was not possible to standardize the procedure across all cohorts. Previous studies in the PACE consortium used this procedure successfully [28–30]. Full information on the methods used within each cohort is reported in the Supplementary Methods and Materials.

### Cognitive skills

Cognitive skills were measured differently across cohorts, depending on available data at cohort-average ages ranging from 4 to 9 years (see Supplementary Material and Methods for more details). Main cognitive scores and subtests were then used to represent overall, verbal and non-verbal cognitive domains.

Overall cognitive skills were measured by:

- the Wechsler Intelligence Scale for Children, 4th edition, (WISC-IV) [31] full-scale IQ score in CHAMACOS and PREDO;
- the Wechsler Intelligence Scale for Children, 3rd edition, (WISC-III) [32] full-scale IQ score in ALSPAC;
- the Wechsler Preschool and Primary Scale of Intelligence (WPPSI), 3rd edition [33] full-scale IQ score in EDEN and POSEIDON;
- the McCarthy Scales of Children’s Abilities [34] general cognitive index in INMA;
- the Wide Range Assessment of Visual Motor Ability (WRAVMA) test [35] in Project Viva.

Verbal cognitive skills were measured by:

- the Wechsler Intelligence Scale for Children, 4th edition, (WISC-IV) [31] verbal comprehension index in CHAMACOS and PREDO;
- the Wechsler Intelligence Scale for Children, 3rd edition, (WISC-III) [32] verbal IQ scores (derived from the verbal comprehension index and working memory index, combined) in ALSPAC;
- the Wechsler Preschool and Primary Scale of Intelligence (WPPSI), 3rd edition [33] verbal IQ score in EDEN and POSEIDON;
- the McCarthy Scales of Children’s Abilities [34] verbal index in INMA;
- the Kaufman Brief Intelligence Test 2nd edition (KBIT-II) [36] verbal subtest in Project Viva.

Non-verbal cognitive skills were measured by:

- the Wechsler Intelligence Scale for Children, 4th edition, (WISC-IV) [31] perceptual reasoning index in CHAMACOS and PREDO;
- the Wechsler Intelligence Scale for Children, 3rd edition, (WISC-III) [32] performance IQ scores (derived from the perceptual organization index and processing speed index, combined) in ALSPAC;
- the Wechsler Preschool and Primary Scale of Intelligence (WPPSI), 3rd edition [33] performance IQ score in EDEN and POSEIDON;
- the McCarthy Scales of Children’s Abilities [34] perceptual-performance index in INMA;
- the Kaufman Brief Intelligence Test 2nd edition (KBIT-II) [36] non-verbal subtest in Project Viva;
- the Snijders-Oomen non-verbal intelligence tests, revised (SON-R) [37], in Generation R.

The average cognitive skills scores across cohort methylation subsamples are reported in Supplementary Table ST1. Since the distribution and the scores used differed across the cohort methylation subsamples, the cognitive scores were transformed into standardized *z*-scores. Full information on the methods used within each cohort is reported in Supplementary Material and Methods.

### Epigenome-wide association study (EWAS)

Prior to the analyses, a data analysis plan including details of the variables, the models to use, and a sample R code was distributed to the participating cohorts. The analysis plan and the code can be accessed upon request to the corresponding author. Untransformed DNA methylation beta values were used as the exposure variable. Extreme outliers (>3 × interquartile range, either side of the 25th and 75th percentiles) were removed.

The effect of DNA methylation at birth on childhood cognitive skills was estimated by linear regression models (*lm*() option in R) within each cohort for each CpG site individually. The main models included the covariates: child age at cognitive testing, sex, maternal age at delivery, maternal education (cohort-specific definition), birth weight, gestational age at birth, maternal smoking status during pregnancy (any smoking compared to no smoking), parity at delivery (1 or more previous children compared to none), batch covariates (cohort-specific definition) and proportions of seven blood cell types estimated using the Houseman algorithm using a published reference dataset for cord blood [38]. The covariates chosen for the main model were those that maximized the sample size as they were available in all cohorts.

As sensitivity analyses, three other models were run including other covariates in cohorts with the relevant data available: main model covariates + paternal education, main model covariates with maternal IQ in place of maternal education, and main model covariates + the first ten principal components from children’s genomic data. Maternal IQ was not included in the main model since not all the cohorts had this variable available. We excluded maternal education in the model with maternal IQ to avoid multicollinearity due to the high correlation between these two variables. Genomic PCs were not included in the main model as they were not available for all cohorts. Each model was run for overall, verbal and non-verbal cognitive skills. Details of the EWAS run within each cohort and specific information on the covariates used are in Supplementary Material and Methods. A summary of the models and the corresponding sample sizes is available in Table 1.

**Table 1:** Overview of the studies participating in the meta-analysis.

Cohort	Cognitive outcome	Age (years)	Overall cognitive abilities (*N*)				Verbal cognitive abilities (*N*)				Non-verbal cognitive abilities (*N*)			
			Main	Paternal education	Maternal IQ	PCs	Main	Paternal education	Maternal IQ	PCs	Main	Paternal education	Maternal IQ	PCs
ALSPAC	WISC-III	9	780	739	–	684	784	743	–	687	783	742	–	687
CHAMACOS	WISC-IV	7	175	175	175	–	175	175	175	–	175	175	175	–
EDEN	WPPSI	6	157	157	–	–	157	157	–	–	157	157	–	–
Generation R	SON-R	6	–	–	–	–	–	–		–	1093	953	1058	1058
INMA	McCarthy	4	319	319	311	280	319	319	311	280	319	319	311	280
POSEIDON	WPPSI	4	199	198	–	198	201	200	–	200	203	202	–	202
PREDO	WISC-IV	9	285	192	–	285	285	192	–	285	285	192	–	285
Project Viva	KBIT-II	8	281	269	279	–	285	273	283	–	285	273	283	–
Total			2196	2049	765	1447	2206	2059	769	1452	3300	3013	1827	2512

The main model was adjusted for child age at cognitive testing, sex, maternal age at delivery, maternal education, birth weight, gestational age at birth, maternal smoking status during pregnancy, parity at delivery, batch covariates and cell type proportions. The other models included also paternal education, maternal IQ (in place of maternal education) and genomic PCs.

### Meta-analysis

The results from the EWAS carried out in each cohort were subjected to an initial screening to check that results were comparable across cohorts using the *QCEWAS* R package [39]. Through visual inspection we assessed similarity of effect size distribution across cohorts and the presence of a linear relationship between precision (1/medianSE) and sample size (sqrt-transformed), as suggested by the package manual. A fixed-effect meta-analysis was then performed for each model and cognitive outcome using the inverse-variance weighted approach in the *metafor* R package [40]. An independent meta-analysis was conducted at a different research institution using the *metasoft* software [41] to confirm the results. Multiple comparisons were taken into account by setting a Bonferroni-corrected threshold of 1.02 × 10^−7^ for the 450 K arrays, which is conservative when taking into account the reduced number of CpGs common to all studies (i.e., excluding outlier and low detection probes and restricting to common probes between the 450 K and EPIC arrays). The number of CpG sites was 379445, 379445, 321854 for overall, verbal and non-verbal cognitive skills, respectively.

As some heterogeneity was expected due to the different studies and methods, we also performed a random effect meta-analysis using the DerSimonian-Laird estimator in the *metafor* R package on the 500 sites that had the lowest *p* values in the fixed effects meta-analysis. Statistical heterogeneity was assessed using the *I*^2^ statistics for each CpG site [42]. As previously recommended [42], heterogeneity was described low, moderate and high around *I*^2^ values of 25%, 50% and 75%, respectively. To explore heterogeneity and since the year of birth varied substantially across the studies, we also performed a random effect meta-regression on the top 500 CpG sites from the fixed effects meta-analysis by including year at the start of the cohort (centered to the mean) as a covariate. To further account for heterogeneity, as cognitive skills were measured using different methods across the studies and at different ages, we also looked for Bonferroni-significant results within each individual study. Finally, we also meta-analyzed the results from the most homogenous group in terms of cognitive skills assessments. This was comprised of the ALSPAC, PREDO and CHAMACOS cohorts, where cognitive skills were assessed using the WISC instrument (3rd or 4th edition) and at similar ages (7–9 years).

To compare our results with the published literature, we looked up in our results the association of cognitive skills and DNA methylation at birth at candidate CpGs based on two previous EWAS studies, the EWAS of cognitive abilities in adulthood [11] and the EWAS of educational attainment [12]. Specifically, from the adult cognitive abilities study we looked up the 2 CpGs that were Bonferroni-significant across all seven cognitive tests and the 43 CpGs that were Bonferroni-significant within specific cognitive tests. From the educational attainment study we looked up the nine CpGs that were significant in adjusted models.

### Differential methylation region (DMR) analysis

To assess the joint effect of blood DNA methylation across different sites on cognitive skills we performed a regional analysis. The results of the meta-analysis for each model were analyzed to detect differential methylation regions using the *dmrff* R package [43]. This method combines EWAS summary statistics from nearby CpG sites while taking into account their correlation by using an approach derived from the inverse-variance weighted meta-analysis. Genomic regions were defined as sets of CpGs less than 500 bp apart, nominal *p* values <0.05 and same sign of effect estimates. This analysis was performed for the main models on overall, verbal and non-verbal scores and for the sensitivity models with further adjustments for paternal education, maternal IQ and genetic PCs.

### Brain DNA methylation and gene expression

Correlations between blood and brain methylation at CpG sites within the DMRs were obtained from two online comparison tools based on methylation data from adult participants (https://epigenetics.essex.ac.uk/bloodbrain/, date accessed 15-07-2020, and https://redgar598.shinyapps.io/BECon/, date accessed 01-12-2020). Since these reference datasets rely on post-mortem brain samples, we could not access data in younger individuals. Brain expression of genes that were in DMRs was examined using the GTEx online portal (https://gtexportal.org/home/, date accessed 15-07-2020) and the Braineac online tool (http://www.braineac.org/).

## Results

### Sample characteristics

All cohorts included in the main meta-analysis (Supplementary Table ST1) included a mix of male and female children (average percentage of females across cohorts ranged from 41 to 54%). Testing for childhood cognitive skills was done at a range of average ages spanning from 4 to 9 years (Table 1 and Supplementary Table ST1). Maternal age at delivery was on average between 26 and 33 years overall. The children were born on average at 39-40 weeks of gestation. Maternal smoking during pregnancy varied across cohorts, with the most prevalent self-report in INMA (27%) and EDEN (25%) and the least prevalent in CHAMACOS (5%) and PREDO (4%). Maternal education was coded differently across the cohorts, with a low percentage of highly educated mothers in ALSPAC and CHAMACOS (20–21%) and highest in Generation R and Project Viva (66–78%). The percentage of children with at least one older sibling also varied across cohorts, ranging from 29 to 64%. Sample characteristics were similar for sensitivity models (Supplementary Tables ST2 and ST3). Average paternal education levels were similar to maternal ones within each cohort. Average maternal IQ was only available for 4 cohorts and it was the lowest in CHAMACOS (mean = 85.7) and the highest in Project Viva (mean = 111.4).

### Epigenome-wide association study meta-analysis

**Fig. 1:**
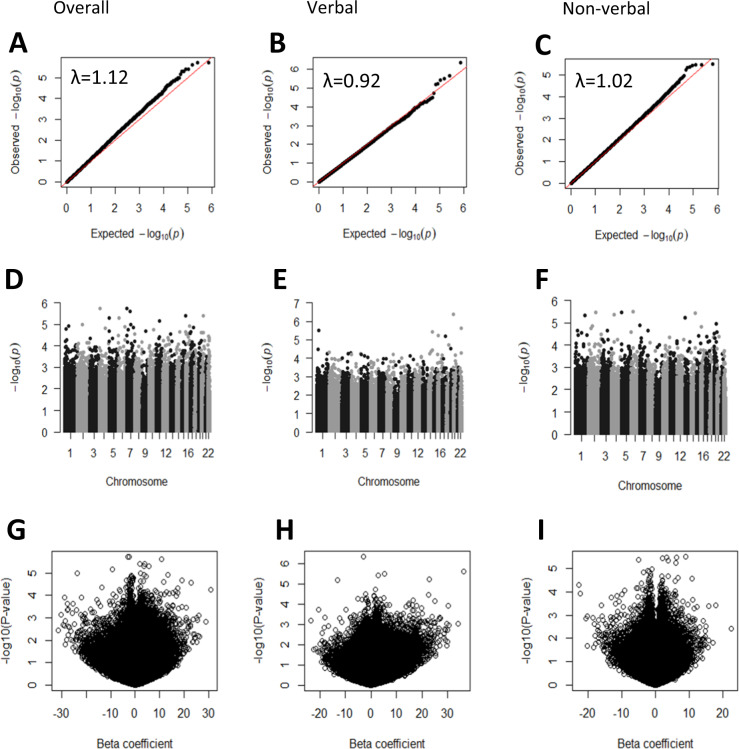
Results from the meta-analysis of epigenome-wide association studies of cognitive skills in childhood and DNA methylation in cord blood. **A**–**C** QQ plots showing the observed vs expected probabilities per CpG site and λ index of genomic inflation. **D**–**F** Manhattan plots showing the −log10(*p* values) at each CpG site according to chromosome location. **G**–**I** Volcano plots showing the effect sizes (difference in IQ *z*-score units per change from 0 to 1 proportion methylated) and probability values for each CpG site. Models were adjusted for age at testing, sex, maternal age at delivery, maternal education, birth weight, gestational age, maternal smoking status during pregnancy, parity, batch covariates and cell proportions.

The results of the meta-EWAS for the main models are plotted in Fig. 1. There was little evidence of an association between cord blood DNA methylation and childhood cognitive skills, either in terms of overall, verbal, or non-verbal scores. No associations passed the Bonferroni-adjusted *p* value cut-off of 1.02 × 10^−7^. The strongest associations, although not significant when taking into account multiple comparisons, are reported in Table 2. One CpG site, cg00573504 which is located in an intergenic region on chromosome 5, showed a similar association with the overall (*β* = 3.71, *p* = 4.98E−06) and the non-verbal cognitive scores (*β* = 3.38, *p* = 3.32E−06), whereas the other top sites (*p* < 10^−5^) were not overlapping across the different cognitive measures. Effect sizes for the top sites were in the range of 0.02–3.6 *z*-score changes per 10% change in methylation, whereas heterogeneity at those sites between contributing studies was low for most sites and moderate for cg10620273 and cg04783204, with *I*^2^ < 60. There was some genomic inflation in the overall score model (*λ* = 1.11). When we performed random effect meta-analysis on the top 500 sites, the effect sizes showed very little change and none of them had *p* values <1.02 × 10^−7^ (see Supplementary Table ST4 for the top sites). The meta-regression analysis showed very little evidence of an effect of year of birth. The meta-regression *p* values for 500 CpGs were all higher than the Bonferroni-corrected threshold 0.05/500 = 10^−4^ (see Supplementary Table ST5 for meta-regression results for the top CpG sites identified in the EWAS at *p* < 10^−5^).

**Table 2:** Top CpG sites (*p*_meta_ < 10^−5^), not significant after Bonferroni correction for multiple comparisons, from the meta-analysis of epigenome-wide association studies of overall, verbal and non-verbal cognitive skills in childhood and DNA methylation in cord blood.

	CpG site	*N*	Beta^a^	SE	*p* value^b^	*I*^2c^	Chr.	Position	Gene^d^
Overall	cg05827775	2193	−3.05	0.64	1.84E−06	0	4	9762166	
	cg00213080	2190	−2.44	0.51	1.89E−06	0	7	6204521	*CYTH3*
	cg26599274	2181	10.83	2.30	2.46E−06	15.1	7	66205733	*RABGEF1*
	cg23789148	2190	2.03	0.44	3.88E−06	0	15	97321146	
	cg18622281	2188	3.96	0.86	3.91E−06	13.8	20	43977112	*SDC4*
	cg00573504	2181	3.71	0.81	4.98E−06	0	5	1123809	
	cg09535605	2174	5.57	1.22	5.09E−06	5.1	6	17281327	*RBM24*
	cg21735491	2190	−9.38	2.09	6.77E−06	0	11	66749665	
	cg18075761	2152	−23.68	5.36	9.84E−06	0	7	75617028	*TMEM120A*
Verbal	cg03568675	2201	−3.00	0.59	4.36E−07	12.3	20	979279	*RSPO4*
	cg12361663	2190	36.38	7.70	2.30E−06	0	22	38142561	*TRIOBP*
	cg17223866	2188	5.21	1.12	3.14E−06	0	1	44794158	*ERI3*
	cg11005998	2199	2.94	0.64	3.87E−06	0	14	101514642	*MIR655*
	cg10620273	2200	22.72	5.00	5.65E−06	51.0	16	3096488	*MMP25*
	cg16047144	2200	−13.16	2.92	6.33E−06	0	17	62097953	*ICAM2*
Non-verbal	cg04783204	3286	8.90	1.91	3.11E−06	53.3	6	44191600	*SLC29A1*
	cg04229103	3278	6.34	1.36	3.31E−06	0	2	145090268	*GTDC1*
	cg00573504	3282	3.38	0.73	3.32E−06	0	5	1123809	
	cg25990848	3276	2.07	0.45	3.59E−06	17.8	14	105517573	*GPR132*
	cg08529049	3254	−4.95	1.08	4.30E−06	25.6	4	48988038	*CWH43*
	cg03332597	3297	3.83	0.84	4.80E−06	37.3	1	185125704	*C1orf25*
	cg07489869	3256	5.94	1.31	5.85E−06	1.8	13	20806730	*GJB6*

^a^Beta coefficient from the regression indicating the change in IQ score standard deviations per change in methylation proportion from 0 to 1. Models were adjusted for age at testing, sex, maternal age at delivery, maternal education, birth weight, gestational age, maternal smoking status during pregnancy, parity, batch covariates and cell proportions.

^b^Unadjusted *p* value.

^c^Heterogeneity statistics.

^d^Gene annotation from the Illumina 450 K manifest file.

The results of the sensitivity models are summarized in Supplementary Figs. SF1 and SF2 and in Supplementary Tables ST6–ST8. None of these models revealed associations with cognitive skills at *p* values lower than 1.02 × 10^−7^. Regarding the top sites from the main model (Supplementary Table ST9), additionally adjusting for paternal education, maternal IQ or genomic PCs did not substantially change the results.

When EWAS results for each cohort were examined individually across the main models using the same *p* value cut-off of 1.02 × 10^−7^ which takes multiple comparisons across the total number of CpGs in the array within each cohort, there was evidence of association of lower non-verbal cognitive skills with DNA methylation at cg26664492 on chromosome 8 (intergenic location) in the ALSPAC cohort (*p* = 8.72 × 10^−8^) (Supplementary Fig. SF3). When we performed meta-analysis across ALSPAC, PREDO and CHAMACOS (WISC assessment of cognitive skills at age 7–9) we did not observe associations at *p* values <1.02 × 10^−7^ (see Supplementary Table ST10 for top sites).

Using the results of the main models, we looked up findings from a recent EWAS meta-analysis of adult blood cells DNA methylation and cognitive abilities in adulthood. The two main CpG sites from the EWAS meta-analysis did not replicate in our study (*p* value >0.05). Amongst the 43 CpGs that were significant within the different cognitive measures reported in Supplementary Table 3 in the cognitive abilities EWAS study [11], we were able to replicate the association at cg17759224 (intergenic region on chromosome 1) methylation in cord blood with non-verbal scores (nominal *p* = 0.00065 lower than *p* = 0.05/34 = 0.001), out of the 34 sites that were available in our meta-analysis (Supplementary Table ST11). Since cognitive development is strongly associated with educational attainment [6], we also checked if cord blood DNA methylation was associated with childhood cognitive skills at CpG sites previously found to be linked to educational attainment [12]. None of the 9 CpG sites from the educational attainment EWAS had cord blood DNA methylation levels associated with childhood cognitive outcomes in our meta-analysis (Supplementary Table ST12).

### DMR analysis

We performed a DMR analysis to investigate the association of DNA methylation at clusters of CpG sites with cognitive skills and found little evidence of clusters in the main models across the cognitive scores (no regions at corrected *p* < 0.05). In the sensitivity model adjusted for maternal IQ (3 cohorts for overall and verbal cognitive skills, 4 cohorts for non-verbal cognitive skills), methylation within a region comprising 5 CpG sites on chromosome 6 was associated with slightly higher non-verbal scores (DMR adjusted *p* value = 0.002, Supplementary Table ST13). Even considering the multiple tests across the main model for non-verbal and the three sensitivity models with further adjustments, this *p* value was lower than a Bonferroni-corrected *p* value of 0.05/4 = 0.0125. The heterogeneity at all five sites was low for the maternal IQ model (*I*^2^ = 0–5). The positive association between methylation and cognitive skills was similar in the main model and in other sensitivity models, although attenuated, with heterogeneity increased to moderate in some of the sites (*I*^2^ = 9–49) (Supplementary Table ST14). A search within two mQTL databases (www.mqtldb.org and mqtldb.godmc.org.uk/) showed only *trans*-mQTLs for these CpG sites, therefore we could not perform any Mendelian randomization analyses to investigate if these associations are causal.

### Brain DNA methylation and gene expression

The DMR is located within the *DUSP22* gene, a phosphatase that is expressed across examined tissues including blood and several brain areas (Supplementary Fig. SF4). DNA methylation in blood within this region highly correlates with brain methylation at all CpGs across all brain regions and across the two datasets interrogated, with *r* > 0.9, *p* < 10^−32^ in the prefrontal cortex, entorhinal cortex, cerebellum and superior temporal gyrus (Table 3), and *r* values of 0.4–0.8, reaching the 90th percentile, for BA10, BA20 and BA7 (Supplementary Fig. SF5).

**Table 3:** Correlation of DNA methylation levels between brain and blood in adult samples (*N* = 71–75, 40–105 years old) in the *DUSP22* differentially methylated region (https://epigenetics.essex.ac.uk/bloodbrain/).

	Prefrontal cortex		Entorhinal cortex		Cerebellum		Superior temporal gyrus	
CpG	*r*	*p* value	*r*	*p* value	*r*	*p* value	*r*	*p* value
cg03395511	0.981	3.83e−53	0.965	5.05e−42	0.984	6.30e−57	0.975	5.60e−47
cg07332563	0.946	5.55e−37	0.940	7.93e−34	0.951	4.76e−39	0.927	3.38e−31
cg15383120	0.971	1.67e−46	0.939	9.17e−34	0.961	2.26e−42	0.937	2.59e−33
cg18110333	0.986	6.57e−58	0.981	7.78e−51	0.986	4.74e−58	0.970	4.82e−44
cg21548813	0.986	5.33e−58	0.975	7.80e−47	0.988	9.75e−61	0.986	2.11e−55

## Discussion

We have performed the largest EWAS of cognitive skills in cord blood, by running individual EWAS in eight cohorts and combining the results through meta-analysis. We hypothesized that, at birth, we would be able to identify methylation variation that was associated with variation in cognitive skills in childhood. Overall, the evidence at single CpG sites was weak across all models to confirm an association. In one region spanning 5 CpGs on chromosome 6 methylation was positively correlated with small increases in non-verbal scores after adjusting for maternal IQ. This association was revealed only in a subgroup sensitivity analysis (3–4 cohorts instead of 7–8). Cord blood DNA methylation in this region was highly correlated with brain methylation, although we could not identify cis-mQTLs to perform further analyses to establish if methylation in this region is causal to variation in cognitive skills.

The main strength of this study is the large sample size achieved by analyzing data from eight cohorts and combining the results in a meta-analysis allowing to identify only robust results. By using the same protocol and script in all the cohorts we have reduced bias due to heterogeneity.

Our study also has some limitations. Cognitive skills were assessed using different instruments across the cohorts investigated. Although Wechsler scales were used in five of the eight cohorts and despite the evidence of strong correlations between composite measures from cognitive tests [44–46], the different tests used could have contributed to the heterogeneity and reduced precision in our estimates. The covariates also differed across cohorts in the way they were measured, for instance in terms of maternal education and smoking. There was also some heterogeneity in terms of year of birth across cohorts, with some starting and ending in the 90s, others in the 00s and others across both decades. However, the meta-regression results did not show a strong effect of year of birth. Although the sample size was large, some cohorts could not participate in some of the models, such as Generation R only having data on non-verbal skills and ALSPAC not having maternal IQ data. Our study consisted mostly of participants that identified themselves as white and lived either in Europe or the US. Therefore, the results of this study are not generalizable globally to other ethnic groups and countries. Moreover, the 450 K methylation arrays only capture 2% of the CpGs in the human genome.

Despite the limitations, the lack of association of DNA methylation at single CpGs at birth with cognitive skills in childhood suggests that DNA methylation does not capture prenatal influences (genetic and environmental) on cognitive development, unlike ADHD and social communication development. This denotes a specificity of DNA methylation at birth for certain neurodevelopmental pathways. Our study replicated the association of one of the 43 subthreshold CpG sites identified in a previous EWAS of cognitive skills in adulthood [11], but none of the nine sites found in an EWAS of educational attainment [12]. In those previous studies, both carried out in adulthood, blood cells methylation patterns seemed to be an indication of lifestyle characteristics such as smoking and BMI. Since our study looked for methylation patterns at birth, we would not necessarily expect to see the effect of direct exposures that would occur later in life. However, maternal smoking is highly associated with DNA methylation at birth [28] and since maternal smoking was included as a covariate in the current analyses, we cannot rule out effects of maternal smoking on cognitive skills via DNA methylation at birth. Further studies using formal mediation models should verify this. If our null results are true, our study suggests that the relationship between cognitive skills and blood DNA methylation seen previously are reflective of exposures after birth, rather than in the prenatal period. Another explanation is that blood methylation marks arise from a gene-environment interaction and they appear only later in life, due to the cumulative effect of environmental exposures that are moderated by genetic variation.

It is also possible that the effect of prenatal exposures on childhood cognitive skills are associated with brain DNA methylation patterns that are not captured by cord blood DNA methylation. Largely due to the inaccessibility of brain tissue, most molecular studies of brain-related traits and disorders rely on blood samples, including cord blood in studies of children at birth. Although DNA methylation patterns are often tissue-specific [47], there are strong cross-tissue correlations at specific sites, and studies on blood-brain correspondence allow us to make comparisons that are relevant to brain phenotypes [48–50]. Moreover, despite blood not being the main target tissue for neurodevelopmental conditions, epigenetic associations have been found in peripheral blood e.g., for schizophrenia and autism [51, 52] and correspondence with brain is not a prerequisite for functionally-relevant DNA methylation changes, for example, where immune-related effects on the brain are potential mechanisms. Additionally, peripheral tissue can still serve as a biomarker, when stable signals are identified.

The differentially methylated region on chromosome 6 is located in the *DUSP22* gene, coding for a phosphatase expressed ubiquitously, including in blood and across brain regions. From previously published data [53], there is evidence that, in whole blood, DNA methylation within this region is associated with increased *DUSP22* expression (Supplementary Table ST15). We also observed that DNA methylation correlates highly between peripheral blood and brain tissue across two different datasets and across brain regions. Although we could only investigate brain-blood correlation in adulthood, we expect some correlation to be present at earlier ages. Moreover, methylation at the *DUSP22* gene in brain tissue has been previously implicated in schizophrenia, Parkinson’s and Alzheimer’s disease, albeit in different directions depending on the brain region investigated [54–56]. More generally, dual-specific phosphatases, including DUSP22, are implicated in a number of neural functions, as shown by several in vivo and in vitro preclinical studies across species, and in a range of mental and neurological disorders (see for review An et al. [57]). These findings, altogether, suggest that DNA methylation may affect brain functioning via changes in *DUSP22* expression. However, in our study this association was observed in only one of the sensitivity models, with a smaller sample size than the other models and without adjustment for maternal education. Although the association in the other models was in the same direction, the evidence was much weaker, suggesting that this association might be sample-specific or confounded by maternal education. Alternatively, this association could be specific for the effect of maternal IQ on offspring’s IQ, which is revealed only when adjusting for maternal IQ. It has been previously shown that maternal IQ affects children cognitive development independently from socioeconomic status and from maternal education [58, 59]. This suggests that maternal IQ could affect the association of methylation at birth with child cognition independently from maternal education and more generally socioeconomic status. Furthermore, this association could be due to residual confounding that was not accounted for in our models (e.g., for aspects of family environment other than socioeconomic status) and needs to be confirmed by replication in independent studies or by using causal inference methods.

In conclusion, we have conducted the largest epigenome-wide scan at birth for cognitive functioning in childhood. Overall, the evidence does not suggest that cord blood DNA methylation at the single CpGs investigated could be an indication of later cognitive skills, either overall, verbal or non-verbal. Most likely, any variation in DNA methylation associated with cognition in peripheral blood arise later in life or are stochastic. Further studies are needed to replicate these results across more ethnically diverse cohorts, in larger samples with more homogenous measurements of cognitive function or in the timing of the cohorts, with data on maternal IQ, and using higher resolution arrays.

## Supplementary information

Supplemental Material

Supplemental Figures

Supplemental Tables

## Data Availability

Meta-analysis results files will be deposited in the EWAS Catalog data repository (http://ewascatalog.org/) upon publication. Individual-level data are available upon request to the cohorts involved and according to their procedures.
