# Supplementary material for: Meta-analysis of epigenome-wide associations between DNA methylation at birth and childhood cognitive skills
Source: Mol Psychiatry. 2022 Feb 10;27(4):2126–35. doi: 10.1038/s41380-022-01441-w (PMC9126809; doi:10.1038/s41380-022-01441-w)
Supplement: Supplementary file 2 — Supplemental Figures [file 41380_2022_1441_MOESM2_ESM.pptx]

## Slide 1
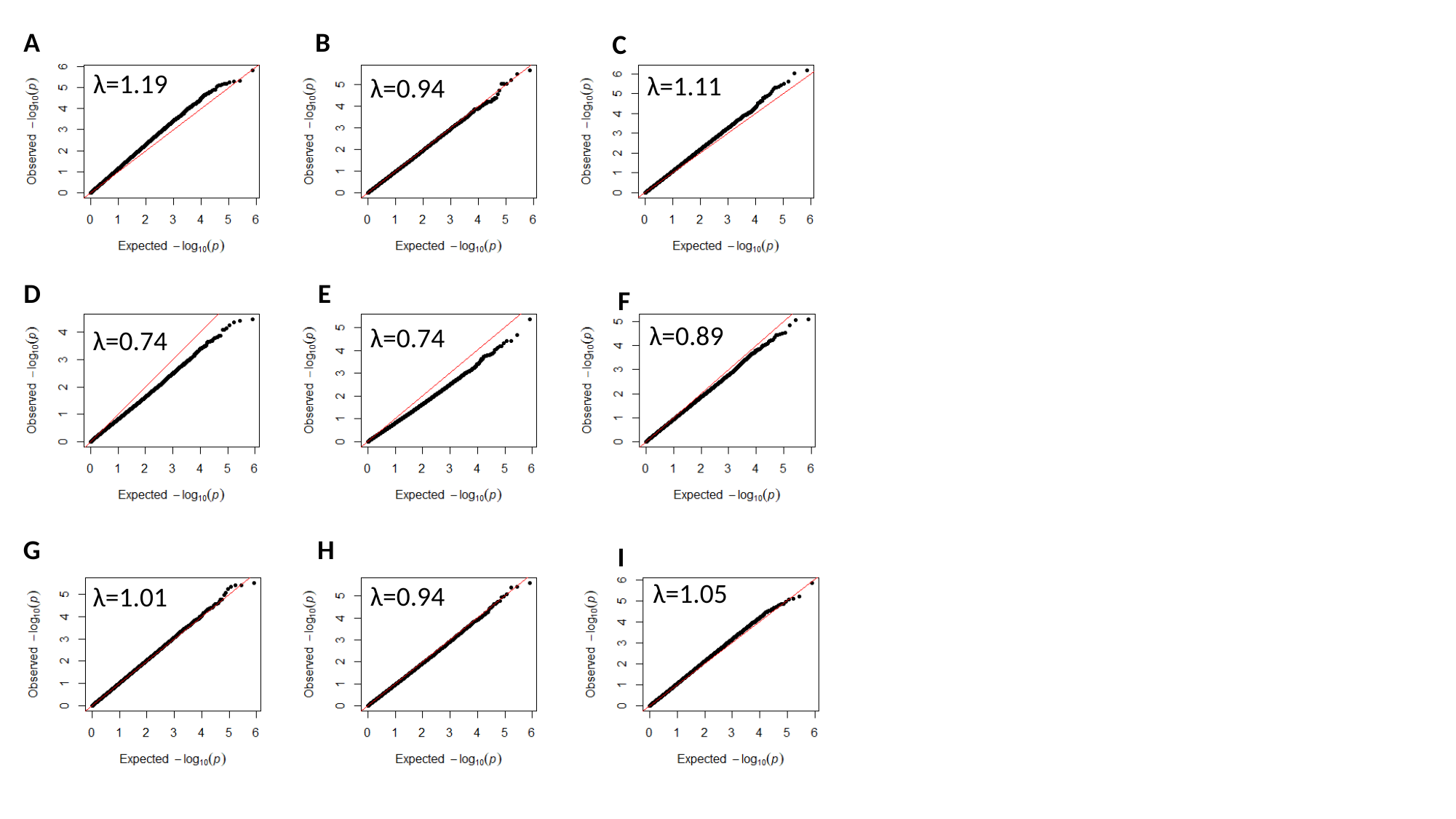

A
B
C
λ=1.19
λ=1.11
λ=0.94
D
E
F
λ=0.89
λ=0.74
λ=0.74
G
H
I
λ=1.05
λ=0.94
λ=1.01

## Slide 2
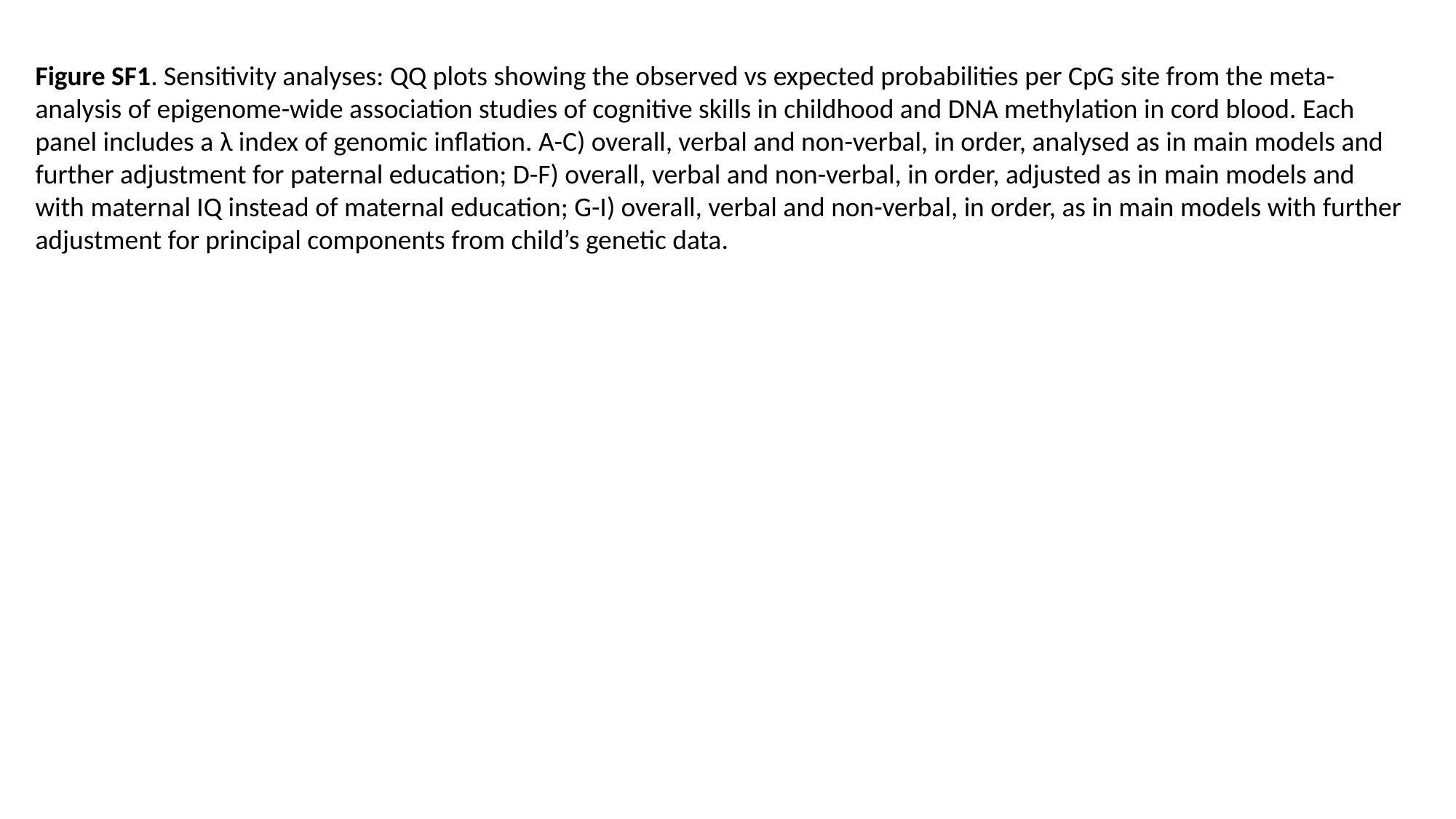

Figure SF1. Sensitivity analyses: QQ plots showing the observed vs expected probabilities per CpG site from the meta-analysis of epigenome-wide association studies of cognitive skills in childhood and DNA methylation in cord blood. Each panel includes a λ index of genomic inflation. A-C) overall, verbal and non-verbal, in order, analysed as in main models and further adjustment for paternal education; D-F) overall, verbal and non-verbal, in order, adjusted as in main models and with maternal IQ instead of maternal education; G-I) overall, verbal and non-verbal, in order, as in main models with further adjustment for principal components from child’s genetic data.

## Slide 3
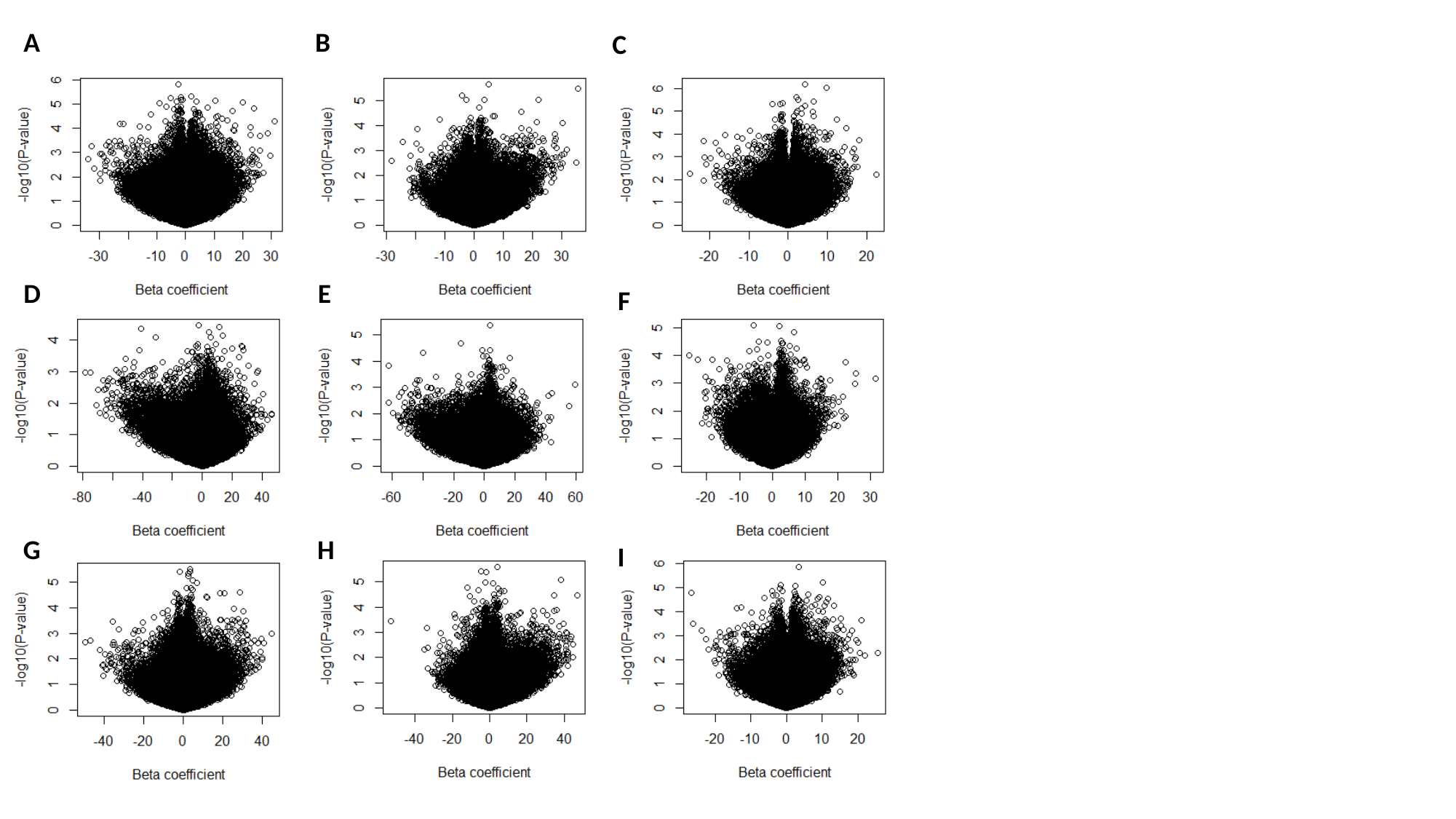

A
B
C
D
E
F
G
H
I

## Slide 4
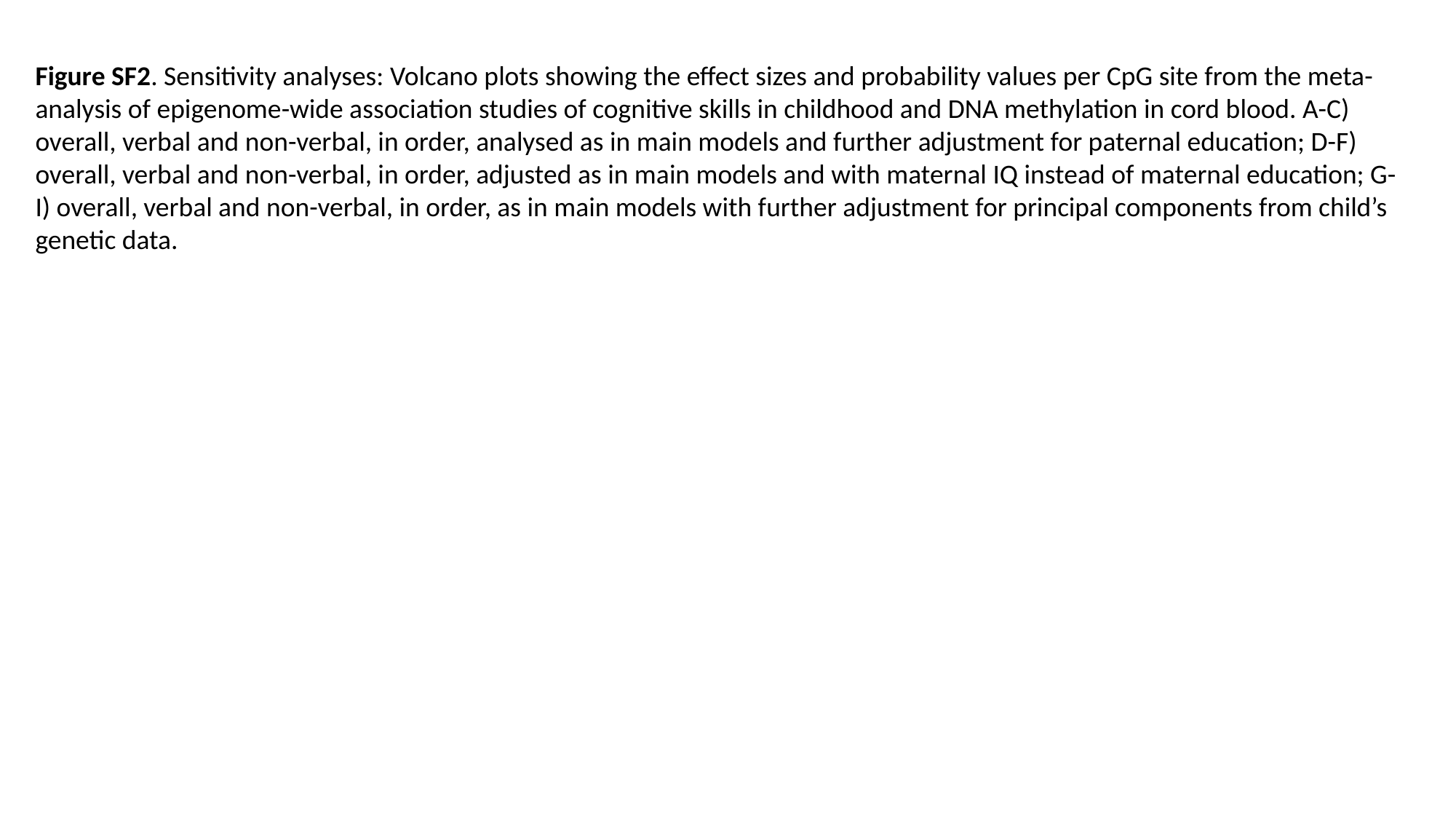

Figure SF2. Sensitivity analyses: Volcano plots showing the effect sizes and probability values per CpG site from the meta-analysis of epigenome-wide association studies of cognitive skills in childhood and DNA methylation in cord blood. A-C) overall, verbal and non-verbal, in order, analysed as in main models and further adjustment for paternal education; D-F) overall, verbal and non-verbal, in order, adjusted as in main models and with maternal IQ instead of maternal education; G-I) overall, verbal and non-verbal, in order, as in main models with further adjustment for principal components from child’s genetic data.

## Slide 5
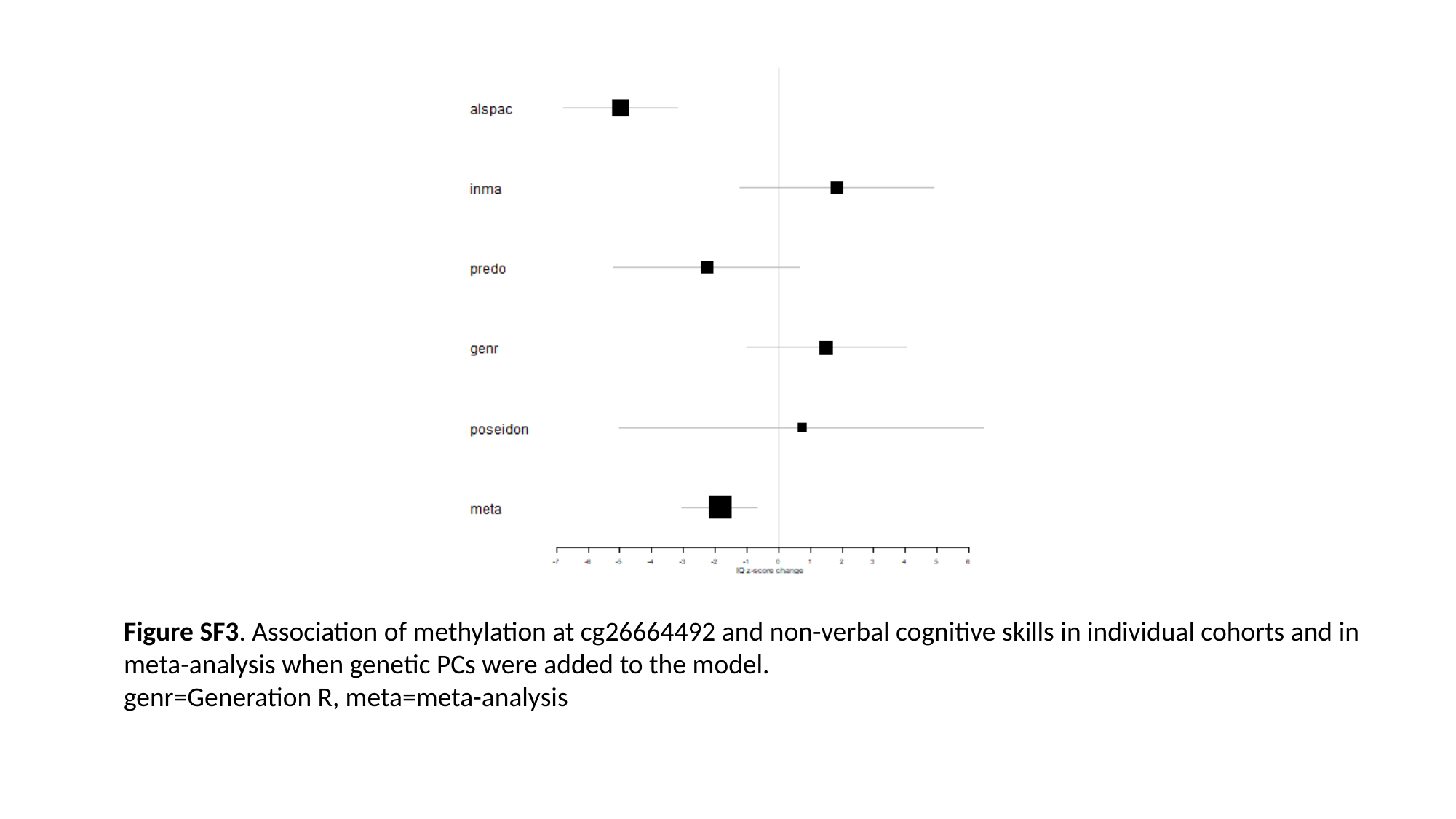

Figure SF3. Association of methylation at cg26664492 and non-verbal cognitive skills in individual cohorts and in meta-analysis when genetic PCs were added to the model.
genr=Generation R, meta=meta-analysis

## Slide 6
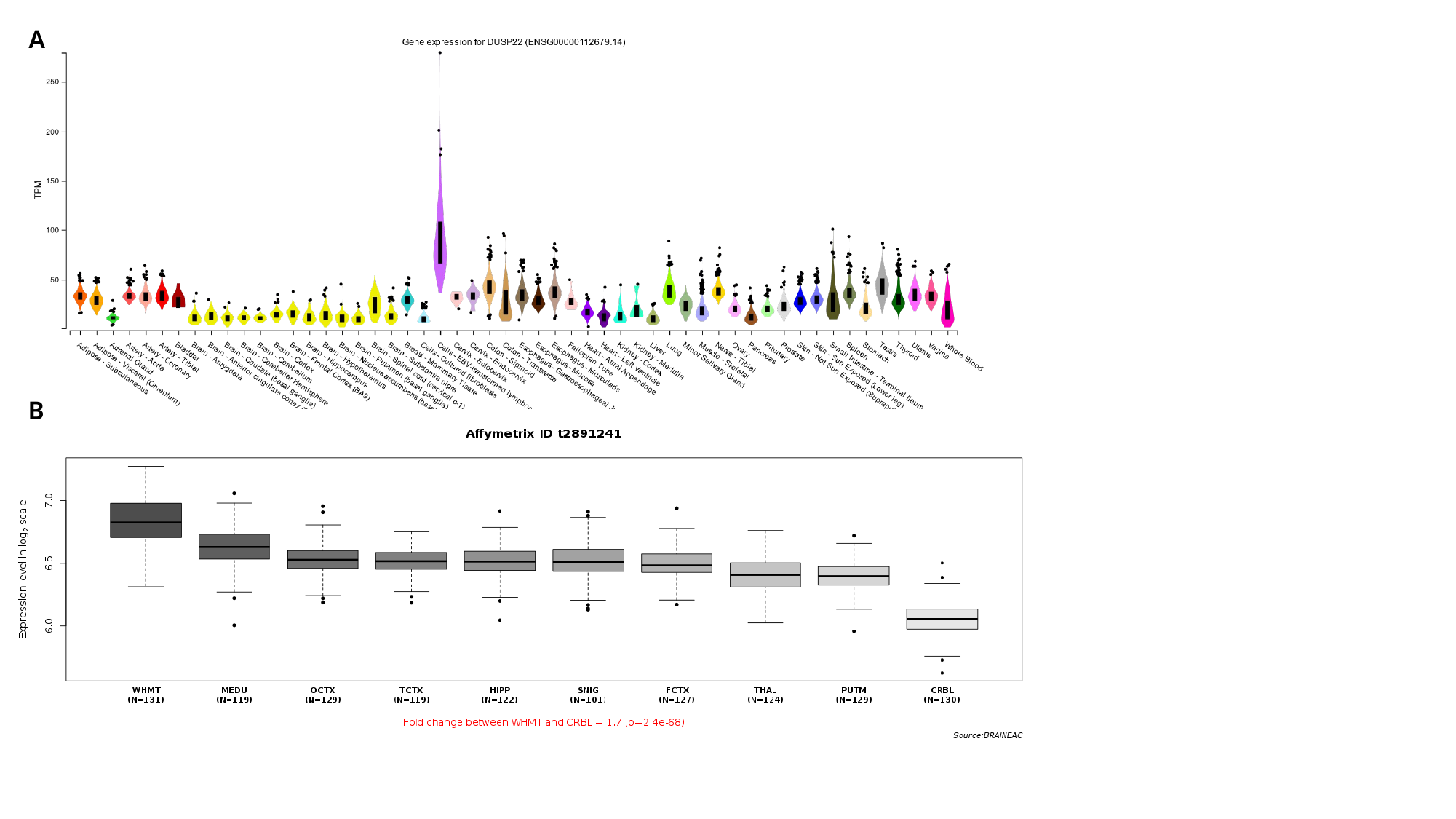

A
B

## Slide 7
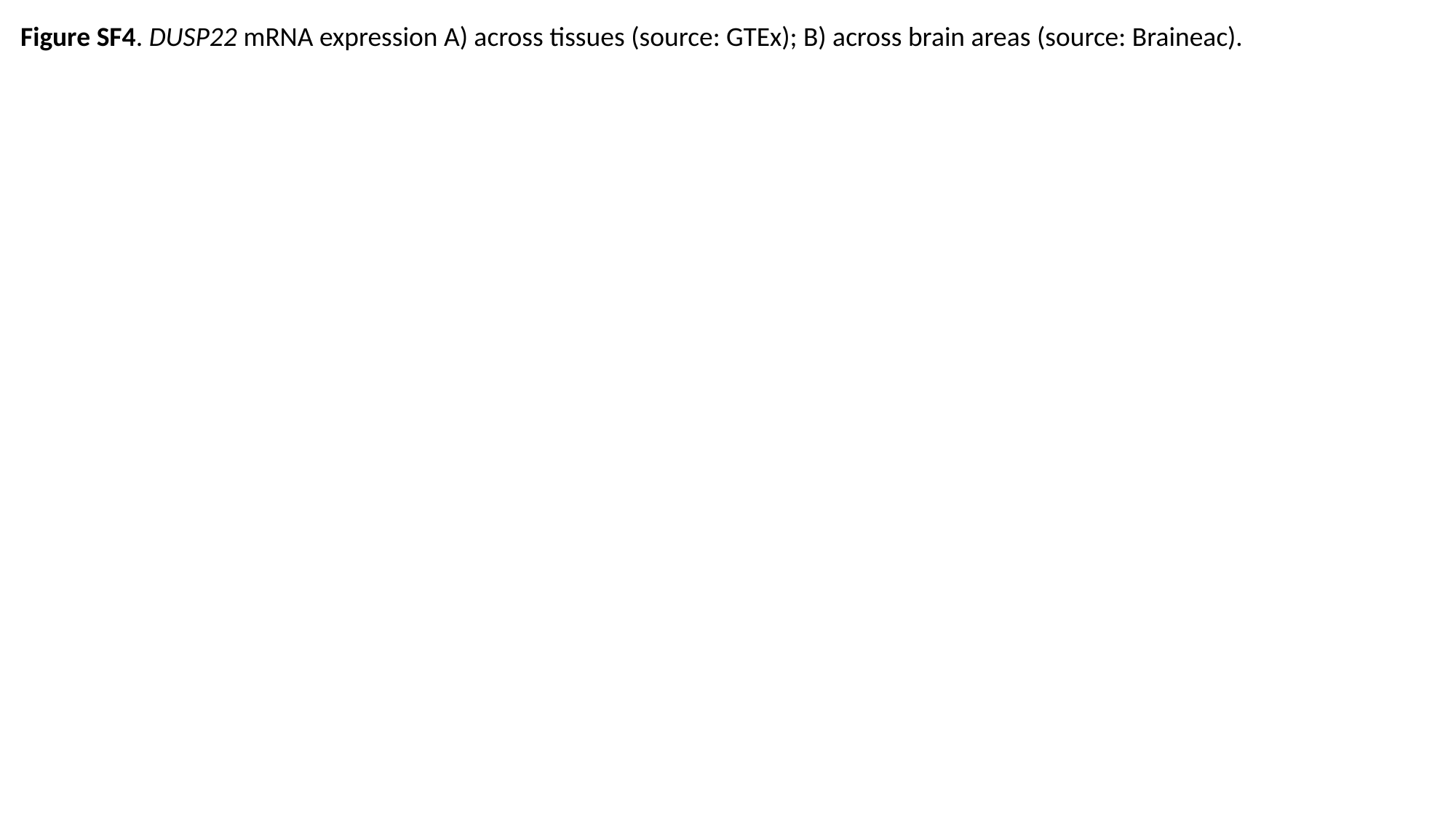

Figure SF4. DUSP22 mRNA expression A) across tissues (source: GTEx); B) across brain areas (source: Braineac).

## Slide 8
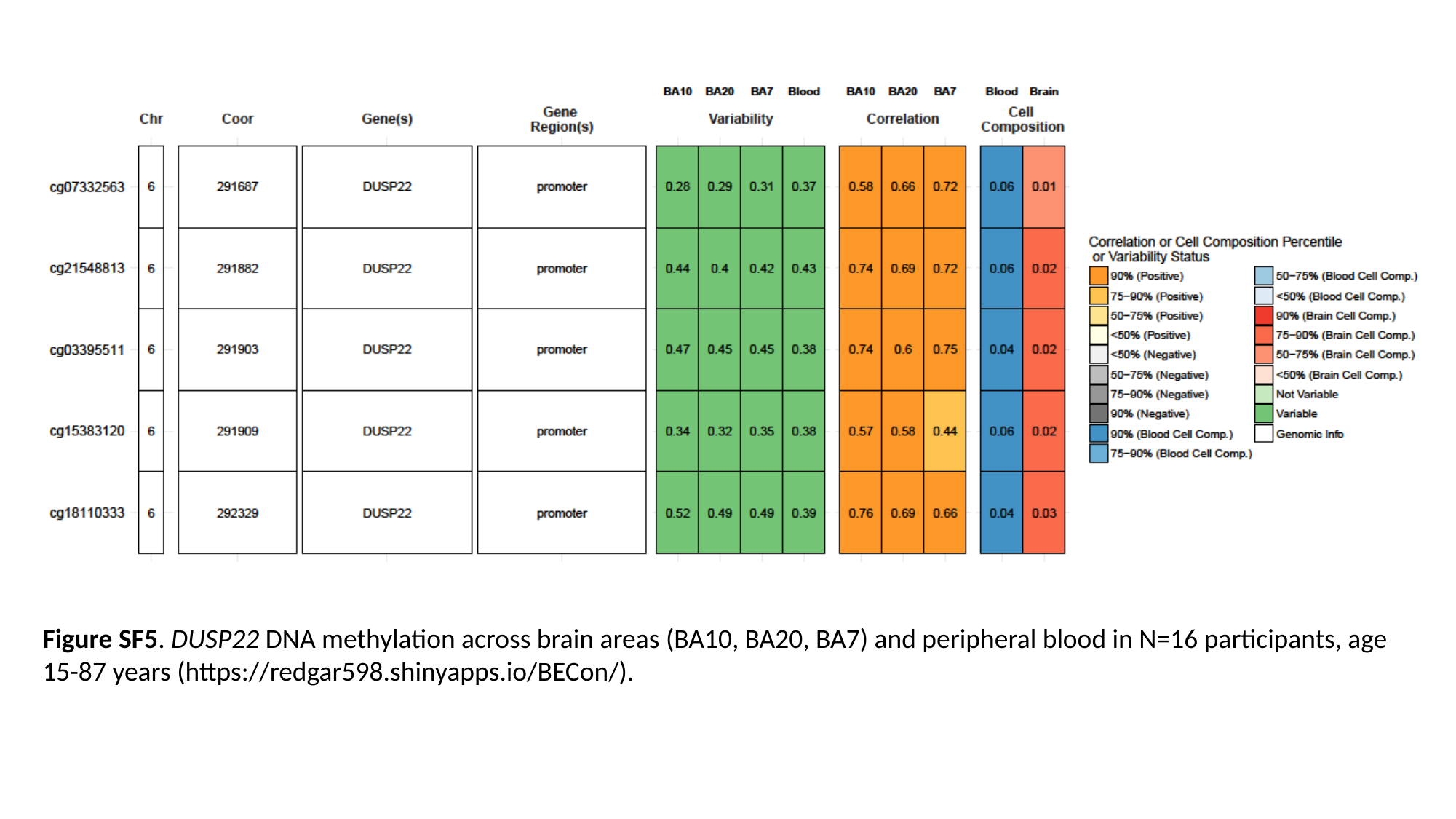

Figure SF5. DUSP22 DNA methylation across brain areas (BA10, BA20, BA7) and peripheral blood in N=16 participants, age 15-87 years (https://redgar598.shinyapps.io/BECon/).
