# Supplementary material for: Meta-analysis of epigenome-wide associations between DNA methylation at birth and childhood cognitive skills
Source: Mol Psychiatry. 2022 Feb 10;27(4):2126–35. doi: 10.1038/s41380-022-01441-w (PMC9126809; doi:10.1038/s41380-022-01441-w)
Supplement: Supplementary file 3 — Supplemental Tables [file 41380_2022_1441_MOESM3_ESM.docx]

**Supplementary Tables**

**Meta-analysis of epigenome-wide associations between DNA methylation at birth and childhood cognitive skills.**

Doretta Caramaschi, Alexander Neumann, Andres Cardenas, Gwen Tindula, Silvia Alemany, Lea Zillich, Giancarlo Pesce, Jari M.T. Lahti, Alexandra Havdahl, Rosa Mulder, Janine F. Felix, Henning Tiemeier, Lea Sirignano, Josef Frank, Stephanie H. Witt, Marcella Rietschel, Michael Deuschle, Karen Huen, Brenda Eskenazi, Tabea Sarah Send, Muriel Ferrer, Maria Gilles, Maria de Agostini, Nour Baïz, Sheryl L. Rifas-Shiman, Tuomas Kvist, Darina Czamara, Samuli T. Tuominen, Caroline L. Relton, Dheeraj Rai, Stephanie J. London, Katri Räikkönen, Nina Holland, Isabella Annesi-Maesano, Fabian Streit, Marie-France Hivert, Emily Oken, Jordi Sunyer, Charlotte A. M. Cecil, Gemma Sharp.

**Supplementary Table ST1**. Sample characteristics across the cohorts used in the meta-analysis EWAS for cognitive abilities (Main models: O= Overall, V=Verbal, NV= Non-Verbal).

|  | **ALSPAC** | | | **CHAMACOS** | | | **EDEN** | | | **Gen. R** | **INMA** | | | **POSEIDON** | | | **PREDO** | | | **Project Viva** | | |
| --- | --- | --- | --- | --- | --- | --- | --- | --- | --- | --- | --- | --- | --- | --- | --- | --- | --- | --- | --- | --- | --- | --- |
|  | **O** | **V** | **NV** | **O** | **V** | **NV** | **O** | **V** | **NV** | **NV** | **O** | **V** | **NV** | **O** | **V** | **NV** | **O** | **V** | **NV** | **O** | **V** | **NV** |
| **N** | 780 | 784 | 783 | 175 | 175 | 175 | 157 | 157 | 157 | 1093 | 319 | 319 | 319 | 199 | 201 | 203 | 285 | 285 | 285 | 281 | 285 | 285 |
| **Cognitive score***  **(mean, SD)** | 108.0  (15.5) | 110.9  (16.4) | 102.4  (16.9) | 104.4  (15.2) | 107.0  (18.0) | 102.3  (16.6) | 102.9 (11.7) | 106.5 (12.0) | 99.1  (13.7) | 106.17  (14.3) | 108.9  (14.4) | 54.0  (9.8) | 53.5  (6.7) | 104.2  (10.5) | 105.6  (10.7) | 101.5  (12.4) | 99.5  (13.1) | 101.0  (14.2) | 99.8  (13.4) | 91.1  (15.7) | 115.2  (13.3) | 107.0  (2.6) |
| **Sex (% females)** | 52 | 52 | 51 | 54 | 54 | 54 | 41 | 41 | 41 | 51 | 48 | 48 | 48 | 56 | 56 | 56 | 49 | 49 | 49 | 50 | 50 | 50 |
| **Child age at test,**  **years mean (SD)** | 8.6  (0.2) | 8.6  (0.2) | 8.6  (0.2) | 7.1  (0.2) | 7.1  (0.2) | 7.1  (0.2) | 5.7  (0.1) | 5.7  (0.1) | 5.7  (0.1) | 6.1  (0.3) | 4.5  (0.2) | 4.5  (0.2) | 4.5  (0.2) | 3.7  (0.1) | 3.7  (0.1) | 3.7  (0.1) | 8.7  (0.8) | 8.7  (0.8) | 8.7  (0.8) | 7.8  (0.7) | 7.8  (0.8) | 7.8  (0.7) |
| **Maternal age**  **at delivery,**  **years mean (SD)** | 29.8  (4.4) | 29.8  (4.4) | 29.8  (4.4) | 26.4  (5.3) | 26.4  (5.3) | 26.4  (5.3) | 30.3  (5.0) | 30.3  (5.0) | 30.3  (5.0) | 31.9  (4.1) | 30.4  (4.1) | 30.4  (4.1) | 30.4  (4.1) | 32.0  (5.0) | 32.0  (4.6) | 31.7  (4.6) | 34.0  (5.5) | 34.0  (5.5) | 34.0  (5.5) | 33.3  (4.4) | 33.3  (4.3) | 33.3  (4.3) |
| **Gestational age,**  **weeks mean (SD)** | 39.6  (1.5) | 39.6  (1.5) | 39.6  (1.5) | 39 .0  (1.4) | 39.0  (1.4) | 39.0  (1.4) | 39.5  (1.4) | 39.5  (1.4) | 39.5  (1.4) | 40.2  (1.5) | 39.8  (1.3) | 39.8  (1.3) | 39.8  (1.3) | 39.2  (1.1) | 39.2  (1.2) | 39.2  (1.2) | 39.8  (1.6) | 39.7  (1.6) | 39.7  (1.6) | 39.9  (1.5) | 39.9  (1.5) | 39.9  (1.5) |
| **Smoking in pregnancy**  **(%)** | 13 | 13 | 13 | 5 | 5 | 5 | 25 | 25 | 25 | 20 | 27 | 27 | 27 | 24 | 23 | 24 | 4 | 4 | 4 | 7 | 7 | 7 |
| **Maternal education**  **(N per group or mean**  **and SD)*** | 619 | 622 | 622 | 137 | 137 | 137 | 38 | 38 | 38 | 12 | 78 | 78 | 78 | 16.8  (3.5) | 16.8  (3.5) | 16.8  (3.5) | 0 | 0 | 0 | 62 | 63 | 63 |
|  | 161 | 162 | 161 | 38 | 38 | 38 | 67 | 67 | 67 | 89 | 137 | 137 | 137 |  |  |  | 3 | 3 | 3 | 219 | 222 | 222 |
|  |  |  |  |  |  |  | 52 | 52 | 52 | 260 | 104 | 104 | 104 |  |  |  | 103 | 103 | 103 |  |  |  |
|  |  |  |  |  |  |  |  |  |  | 297 |  |  |  |  |  |  | 72 | 72 | 72 |  |  |  |
|  |  |  |  |  |  |  |  |  |  | 435 |  |  |  |  |  |  | 107 | 107 | 107 |  |  |  |
|  |  |  |  |  |  |  |  |  |  |  |  |  |  |  |  |  | 0 | 0 | 0 |  |  |  |
| **Parity**  **(% at least one sibling)** | 53 | 53 | 53 | 64 | 64 | 64 | 58 | 58 | 58 | 38 | 41 | 41 | 41 | 49 | 49 | 49 | 29 | 29 | 29 | 52 | 52 | 52 |

* In all cohorts except for INMA the scales of the cognitive scores are standardised to the general population with mean=100 and SD of 15. In INMA, the verbal and non-verbal scores are standardised with mean=50 and SD=10. In all analyses, all scores were transformed to z-scores, with mean=0 and SD=1.

** ALSPAC: low= no university degree, high= university degree; CHAMACOS: low= less than high school, high=at least high school; EDEN: low= upper secondary education, middle= post-secondary non-tertiary, high=tertiary; Generation R= continuous education level from low to high (primary, secondary-phase 1, secondary-phase 2, higher-phase 1, higher-phase 2); INMA: low, middle, high; POSEIDON: years of education (mean, SD); PREDO: groups 1-6 (7-9 had N=0) from high to low education (continuous); Project Viva: low= not college graduate, high= college graduate.

**Supplementary Table ST2**. Sample characteristics across the cohorts used in the meta-analysis EWAS for cognitive abilities (Sensitivity models further adjusted for paternal education: O= Overall, V=Verbal, NV= Non-Verbal).

|  | **ALSPAC** | | | **CHAMACOS** | | | **EDEN** | | | **Gen. R** | **INMA** | | | **POSEIDON** | | | **PREDO** | | | **Project Viva** | | |
| --- | --- | --- | --- | --- | --- | --- | --- | --- | --- | --- | --- | --- | --- | --- | --- | --- | --- | --- | --- | --- | --- | --- |
|  | **O** | **V** | **NV** | **O** | **V** | **NV** | **O** | **V** | **NV** | **NV** | **O** | **V** | **NV** | **O** | **V** | **NV** | **O** | **V** | **NV** | **O** | **V** | **NV** |
| **N** | 739 | 743 | 742 | 175 | 175 | 175 | 157 | 157 | 157 | 953 | 319 | 319 | 319 | 198 | 200 | 202 | 192 | 192 | 192 | 269 | 273 | 273 |
| **Sex (% females)** | 51 | 51 | 51 | 54 | 54 | 54 | 41 | 41 | 41 | 52 | 48 | 48 | 48 | 57 | 56 | 56 | 50 | 50 | 50 | 51% | 51% | 51% |
| **Child age at test, years mean**  **(SD)** | 8.6  (0.2) | 8.6  (0.2) | 8.6  (0.2) | 7.1  (0.2) | 7.1  (0.2) | 7.1  (0.2) | 5.7  (0.1) | 5.7  (0.1) | 5.7  (0.1) | 6.1  (0.3) | 4.5  (0.2) | 4.5  (0.2) | 4.5  (0.2) | 44.9  (0.9) | 44.9  (0.9) | 44.9  (0.9) | 8.5  (0.7) | 8.5  (0.7) | 8.5  (0.7) | 7.8 (0.7) | 7.8 (0.7) | 7.8 (0.7) |
| **Maternal age at delivery, years**  **mean (SD)** | 29.8  (4.3) | 29.8  (4.4) | 29.9  (4.4) | 26.4  (5.3) | 26.4  (5.3) | 26.4  (5.3) | 30.3  (5.0) | 30.3  (5.0) | 30.3  (5.0) | 31.8  (4.0) | 30.4  (4.1) | 30.4  (4.1) | 30.4  (4.1) | 31.8  (4.5) | 31.8  (4.5) | 31.8  (4.6) | 33.8  (5.1) | 33.8  (5.1) | 33.8  (5.1) | 33.4 (4.3) | 33.4 (4.3) | 33.4 (4.3) |
| **Gestational age, weeks mean**  **(SD)** | 40  (1.5) | 39.6  (1.5) | 39.6  (1.5) | 39  (1.4) | 39  (1.4) | 39  (1.4) | 39.5  (1.4) | 39.5  (1.4) | 39.5  (1.4) | 40.2  (1.5) | 39.8  (1.3) | 39.8  (1.3) | 39.8  (1.3) | 39.2  (1.2) | 39.2  (1.2) | 39.2  (1.2) | 39.7  (1.7) | 39.7  (1.7) | 39.7  (1.7) | 40.0 (1.5) | 40.0 (1.5) | 40.0 (1.5) |
| **% any smoking in pregnancy** | 12 | 12 | 12 | 5 | 5 | 5 | 25 | 25 | 25 | 20 | 27 | 27 | 27 | 46 | 46 | 47 | 4 | 4 | 4 | 7 | 7 | 7 |
| **Maternal education**  **(N per group or mean and SD)*** | 579 | 582 | 582 | 137 | 137 | 137 | 38 | 38 | 38 | 0 | 78 | 78 | 78 | 16.8  (3.5) | 16.8  (3.5) | 16.8 (3.5) | 0 | 0 | 0 | 58 | 59 | 59 |
|  | 160 | 161 | 160 | 38 | 38 | 38 | 67 | 67 | 67 | 10 | 137 | 137 | 137 |  |  |  | 3 | 3 | 3 | 211 | 214 | 214 |
|  |  |  |  |  |  |  |  |  |  | 73 | 104 | 104 | 104 |  |  |  | 57 | 57 | 57 |  |  |  |
|  |  |  |  |  |  |  |  |  |  | 222 |  |  |  |  |  |  | 51 | 51 | 51 |  |  |  |
|  |  |  |  |  |  |  |  |  |  | 262 |  |  |  |  |  |  | 81 | 81 | 81 |  |  |  |
|  |  |  |  |  |  |  |  |  |  | 386 |  |  |  |  |  |  | 0 | 0 | 0 |  |  |  |
| **Paternal education**  **(N per group or mean and SD)*** | 532 | 536 | 535 | 148 | 148 | 148 | 48 | 48 | 48 | 0 | 111 | 111 | 111 | 16.39 (3.78) | 16.39 (3.78) | 16.39 (3.77) | 0 | 0 | 0 | 72 | 74 | 74 |
|  | 207 | 207 | 207 | 127 | 127 | 127 | 72 | 72 | 72 | 22 | 143 | 143 | 143 |  |  |  | 0 | 0 | 0 | 197 | 199 | 199 |
|  |  |  |  |  |  |  | 37 | 37 | 37 | 100 | 65 | 65 | 65 |  |  |  | 3 | 3 | 3 |  |  |  |
|  |  |  |  |  |  |  |  |  |  | 219 |  |  |  |  |  |  | 22 | 22 | 22 |  |  |  |
|  |  |  |  |  |  |  |  |  |  | 195 |  |  |  |  |  |  | 40 | 40 | 40 |  |  |  |
|  |  |  |  |  |  |  |  |  |  | 417 |  |  |  |  |  |  | 34 | 34 | 34 |  |  |  |
|  |  |  |  |  |  |  |  |  |  |  |  |  |  |  |  |  | 38 | 38 | 38 |  |  |  |
|  |  |  |  |  |  |  |  |  |  |  |  |  |  |  |  |  | 55 | 55 | 55 |  |  |  |
|  |  |  |  |  |  |  |  |  |  |  |  |  |  |  |  |  | 0 | 0 | 0 |  |  |  |
| **Parity (% at least one sibling)** | 54 | 54 | 54 | 38 | 38 | 38 | 58 | 58 | 58 | 37 | 41 | 41 | 41 | 49 | 49 | 49 | 33 | 33 | 33 | 53 | 52 | 52 |

* ALSPAC: low= no university degree, high= university degree; CHAMACOS: low= less than high school, high=at least high school; EDEN: low= upper secondary education, middle= post-secondary non-tertiary, high=tertiary; Generation R= continuous education level from low to high (primary, secondary-phase 1, secondary-phase 2, higher-phase 1, higher-phase 2); INMA: low, middle, high; POSEIDON: years of education (mean, SD); PREDO: groups 1-9 (7-9 for maternal had N=0) from high to low education (continuous); Project Viva: low= not college graduate, high= college graduate.

**Supplementary Table ST3**. Sample characteristics across the cohorts used in the meta-analysis EWAS for cognitive abilities (Sensitivity models adjusted for maternal IQ instead of maternal education: O= Overall, V=Verbal, NV= Non-Verbal).

|  | **CHAMACOS** | | | **Gen. R** | **INMA** | | | **Project Viva** | | |
| --- | --- | --- | --- | --- | --- | --- | --- | --- | --- | --- |
|  | **O** | **V** | **NV** | **NV** | **O** | **V** | **NV** | **O** | **V** | **NV** |
| **N** | 175 | 175 | 175 | 1058 | 311 | 311 | 311 | 279 | 283 | 283 |
| **Sex (% females)** | 54 | 54 | 54 | 51 | 49 | 49 | 49 | 50 | 50 | 50 |
| **Child age at test, years mean**  **(SD)** | 7.1  (0.2) | 7.1  (0.2) | 7.1  (0.2) | 6.1  (0.3) | 4.5  (0.2) | 4.5  (0.2) | 4.5  (0.2) | 7.9  (0.7) | 7.9  (0.8) | 7.9  (0.8) |
| **Maternal age at delivery, years**  **mean (SD)** | 26.4  (5.3) | 26.4  (5.3) | 26.4  (5.3) | 31.9  (4.1) | 30.4  (4.1) | 30.4  (4.1) | 30.4  (4.1) | 33.4  (4.4) | 33.4  (4.4) | 33.4  (4.4) |
| **Gestational age, weeks mean**  **(SD)** | 39  (1.4) | 39  (1.4) | 39  (1.4) | 40.2  (1.5) | 39.8  (1.3) | 39.8  (1.3) | 39.8  (1.3) | 40.0  (1.5) | 40.0  (1.5) | 40.0  (1.5) |
| **% any smoking in pregnancy** | 5 | 5 | 5 | 20 | 27 | 27 | 27 | 7 | 7 | 7 |
| **Maternal IQ score *, mean (SD)** | 85.7  (20.5) | 85.7  (20.5) | 85.7  (20.5) | 102.8  (12.1) | 10.6  (2.8) | 10.6  (2.8) | 10.6  (2.8) | 111.4  (12.3) | 111.4  (12.3) | 111.4  (12.3) |
| **Parity (% at least one sibling)** | 64 | 64 | 64 | 38 | 42 | 42 | 42 | 53 | 52 | 52 |

CHAMACOS= Peabody Vocabulary Test, Generation R= Raven’s Advanced Progressive Matrices Test, INMA= Similarities subtest from the Wechsler Adult Intelligence-3rd edition, Project Viva= Kaufman Brief Intelligence Test 2nd edition

**Supplementary Table ST4.** Results of the random effect meta-analysis for the CpG sites with lowest p-value (<10^-5^) in the fixed effect meta-analysis.

|  | **CpG site** | **Beta**  **fixed** | **S.E.**  **fixed** | **P-value**  **fixed** | **I^2^** | **Beta**  **random** | **S.E.**  **random** | **P-value**  **random** |
| --- | --- | --- | --- | --- | --- | --- | --- | --- |
| **Overall** | cg05827775 | -3.05 | 0.64 | 1.84E-06 | 0 | -3.05 | 0.64 | 1.84E-06 |
|  | cg00213080 | -2.44 | 0.51 | 1.89E-06 | 0 | -2.44 | 0.51 | 1.89E-06 |
|  | cg26599274 | 10.83 | 2.30 | 2.46E-06 | 15.1 | 10.41 | 2.58 | 5.62E-05 |
|  | cg23789148 | 2.03 | 0.44 | 3.88E-06 | 0 | 2.03 | 0.44 | 3.88E-06 |
|  | cg18622281 | 3.96 | 0.86 | 3.91E-06 | 13.7 | 3.76 | 0.99 | 0.0001 |
|  | cg00573504 | 3.71 | 0.81 | 4.98E-06 | 0 | 3.71 | 0.81 | 4.98E-06 |
|  | cg09535605 | 5.57 | 1.22 | 5.09E-06 | 5.1 | 5.63 | 1.32 | 1.90E-05 |
|  | cg21735491 | -9.38 | 2.09 | 6.77E-06 | 0 | -9.38 | 2.09 | 6.77E-06 |
|  | cg18075761 | -23.68 | 5.36 | 9.84E-06 | 0 | -23.68 | 5.36 | 9.84E-06 |
| **Verbal** | cg03568675 | -3.00 | 0.59 | 4.36E-07 | 12.3 | -2.86 | 0.68 | 2.63E-05 |
|  | cg12361663 | 36.38 | 7.70 | 2.30E-06 | 0 | 36.38 | 7.70 | 2.30E-06 |
|  | cg17223866 | 5.21 | 1.12 | 3.14E-06 | 0 | 5.21 | 1.12 | 3.14E-06 |
|  | cg11005998 | 2.94 | 0.64 | 3.87E-06 | 0 | 2.94 | 0.64 | 3.87E-06 |
|  | cg10620273 | 22.72 | 5.00 | 5.65E-06 | 51 | 21.67 | 8.34 | 0.009 |
|  | cg16047144 | -13.16 | 2.92 | 6.33E-06 | 0 | -13.16 | 2.92 | 6.33E-06 |
| **Non-verbal** | cg04783204 | 8.90 | 1.91 | 3.11E-06 | 53.3 | 8.07 | 3.44 | 0.02 |
|  | cg04229103 | 6.34 | 1.36 | 3.31E-06 | 0 | 6.34 | 1.36 | 3.31E-06 |
|  | cg00573504 | 3.38 | 0.73 | 3.32E-06 | 0 | 3.38 | 0.73 | 3.32E-06 |
|  | cg25990848 | 2.07 | 0.45 | 3.59E-06 | 17.8 | 1.99 | 0.65 | 0.002 |
|  | cg08529049 | -4.95 | 1.077 | 4.30E-06 | 25.6 | -5.41 | 1.42 | 0.0001 |
|  | cg03332597 | 3.83 | 0.84 | 4.80E-06 | 37.3 | 3.76 | 1.16 | 0.001 |

**Supplementary Table ST5.** Results of the meta regression for the CpG sites with lowest p-value (<10^-5^) in the fixed effect meta-analysis.

|  | **CpG site** | **Intercept^a^** | **S.E.** | **P-value** | **Beta^b^** | **S.E.** | **P-value** | **I^2^** |
| --- | --- | --- | --- | --- | --- | --- | --- | --- |
| **Overall** | cg05827775 | -2.86 | 0.84 | 0.0007 | 0.03 | 0.12 | 0.83 | 10.2 |
|  | cg00213080 | -1.68 | 0.76 | 0.0265 | 0.12 | 0.09 | 0.17 | 0 |
|  | cg26599274 | 9.92 | 2.89 | 0.0006 | -0.15 | 0.44 | 0.73 | 22.9 |
|  | cg23789148 | 1.95 | 0.53 | 0.0002 | -0.02 | 0.07 | 0.78 | 0 |
|  | cg18622281 | 3.26 | 1.22 | 0.0073 | -0.12 | 0.19 | 0.54 | 22.2 |
|  | cg00573504 | 2.88 | 1.15 | 0.0124 | -0.14 | 0.14 | 0.31 | 0 |
|  | cg09535605 | 5.21 | 1.24 | 2.53E-05 | -0.44 | 0.25 | 0.08 | 0 |
|  | cg21735491 | -9.43 | 2.31 | 4.57E-05 | -0.01 | 0.30 | 0.96 | 0 |
|  | cg18075761 | -20.90 | 5.80 | 0.0003 | 0.95 | 0.76 | 0.21 | 0 |
| **Verbal** | cg03568675 | -2.65 | 0.80 | 0.0009 | 0.04 | 0.12 | 0.72 | 18.8 |
|  | cg12361663 | 35.11 | 8.23 | 1.97E-05 | 0.56 | 1.27 | 0.66 | 0 |
|  | cg17223866 | 5.47 | 1.33 | 3.95E-05 | 0.05 | 0.18 | 0.76 | 7.9 |
|  | cg11005998 | 2.56 | 0.75 | 0.0006 | -0.09 | 0.09 | 0.32 | 0 |
|  | cg10620273 | 23.12 | 7.73 | 0.0028 | -1.77 | 1.24 | 0.15 | 41.7 |
|  | cg16047144 | -13.10 | 2.94 | 8.13E-06 | 0.07 | 0.47 | 0.87 | 0 |
| **Non-verbal** | cg04783204 | 8.00 | 2.78 | 0.0040 | -1.18 | 0.54 | 0.03 | 31.4 |
|  | cg04229103 | 6.32 | 1.36 | 3.49E-06 | -0.39 | 0.34 | 0.26 | 0 |
|  | cg00573504 | 2.81 | 0.87 | 0.0013 | -0.14 | 0.12 | 0.24 | 0 |
|  | cg25990848 | 1.82 | 0.80 | 0.0235 | -0.07 | 0.11 | 0.52 | 18.6 |
|  | cg08529049 | -5.64 | 1.62 | 0.0005 | 0.00 | 0.27 | 0.99 | 35.1 |
|  | cg03332597 | 3.46 | 1.09 | 0.0016 | -0.28 | 0.20 | 0.16 | 26.5 |

^a^ Intercept of meta-regression: change in cognitive score standard deviations per methylation change from unmethylated to fully methylated at average year (2000).

^b^ Effect of years on meta-regression: change in the association between cognitive score and methylation per additional year.

**Supplementary Table ST6**. Top CpG sites (p-value <10^-5^) from the meta-analysis of epigenome-wide association studies of overall, verbal and non-verbal cognitive abilities in childhood and DNA methylation in cord blood with further adjustment for paternal education.

|  | **CpG site** | **N** | **Beta**^a^ | **S.E.** | **P-value** ^b^ | **I^2^** ^c^ | **Chr.** | **Position** | **Gene** ^d^ |
| --- | --- | --- | --- | --- | --- | --- | --- | --- | --- |
| **Overall** | cg00213080 | 2043 | -2.48 | 0.52 | 1.48E-06 | 0 | 7 | 6204521 | *CYTH3* |
|  | cg23789148 | 2043 | 2.12 | 0.46 | 4.60E-06 | 0 | 15 | 97321146 |  |
|  | cg03931865 | 2043 | -1.64 | 0.36 | 5.28E-06 | 41.1 | 16 | 47048107 |  |
|  | cg23336139 | 2038 | -5.18 | 1.14 | 5.78E-06 | 46.3 | 12 | 1.18E+08 | *TESC* |
|  | cg14848685 | 2043 | -1.69 | 0.38 | 6.71E-06 | 0 | 6 | 1.39E+08 | *KIAA1244* |
|  | cg03493774 | 2049 | -1.11 | 0.25 | 6.74E-06 | 0 | 14 | 92879474 | *SLC24A4* |
|  | cg26599274 | 2034 | 10.58 | 2.36 | 7.17E-06 | 0 | 7 | 66205733 | *RABGEF1* |
|  | cg05827775 | 2046 | -2.95 | 0.66 | 7.73E-06 | 17.1 | 4 | 9762166 |  |
|  | cg18622281 | 2041 | 3.90 | 0.87 | 7.76E-06 | 5.8 | 20 | 43977112 | *SDC4* |
|  | cg13775913 | 2038 | 20.11 | 4.50 | 8.13E-06 | 0 | 17 | 7297869 | *PLSCR3* |
|  | cg21735491 | 2043 | -9.20 | 2.08 | 9.38E-06 | 0 | 11 | 66749665 |  |
| **Verbal** | cg09178369 | 2045 | 4.83 | 1.02 | 2.20E-06 | 39.9 | 6 | 7828165 | *BMP6* |
|  | cg12361663 | 2043 | 35.71 | 7.67 | 3.22E-06 | 0 | 22 | 38142561 | *TRIOBP* |
|  | cg05083414 | 2037 | -4.17 | 0.92 | 6.32E-06 | 0 | 4 | 2627039 | *FAM193A* |
|  | cg10620273 | 2053 | 22.04 | 4.97 | 9.19E-06 | 55.8 | 16 | 3096488 | *MMP25* |
|  | cg03568675 | 2054 | -2.85 | 0.64 | 9.28E-06 | 2.0 | 20 | 979279 | *RSPO4* |
|  | cg12961010 | 2049 | 3.58 | 0.81 | 9.50E-06 | 0 | 12 | 132938327 |  |
| **Non-verbal** | cg03332597 | 3011 | 4.30 | 0.86 | 6.49E-07 | 31.0 | 1 | 185125704 | *C1orf25* |
|  | cg04783204 | 3001 | 9.76 | 1.99 | 8.97E-07 | 56.3 | 6 | 44191600 | *SLC29A1* |
|  | cg25990848 | 2991 | 2.18 | 0.46 | 2.45E-06 | 0 | 14 | 105517573 | *GPR132* |
|  | cg21885231 | 3013 | 2.56 | 0.55 | 3.06E-06 | 0 | 7 | 1453743 |  |
|  | cg04229103 | 2991 | 6.58 | 1.42 | 3.74E-06 | 0 | 2 | 145090268 | *GTDC1* |
|  | cg14255471 | 2999 | -1.45 | 0.32 | 4.46E-06 | 42.6 | 14 | 185125704 |  |
|  | cg07216133 | 3003 | -2.10 | 0.46 | 4.80E-06 | 0 | 6 | 44191600 | *RREB1* |
|  | cg16355591 | 2980 | -3.99 | 0.87 | 4.92E-06 | 0 | 16 | 105517573 |  |
|  | cg17935281 | 2967 | 4.21 | 0.93 | 5.84E-06 | 30 | 3 | 1453743 | *SEMA5B* |
|  | cg00573504 | 2995 | 3.39 | 0.76 | 7.53E-06 | 0 | 5 | 145090268 |  |
|  | cg21950196 | 3013 | 2.22 | 0.50 | 9.79E-06 | 0 | 1 | 106660938 | *GUK1* |

^a^ Beta coefficient from the regression indicating the change in IQ score standard deviations per per unit change in proportion methylation. Models were adjusted also for age at testing, sex, maternal age at delivery, maternal education, birthweight, gestational age, maternal smoking status during pregnancy, parity, batch covariates and cell proportions

^b^ Uncorrected p-value

^c^ Heterogeneity statistics

^d^ Gene annotation from the Illumina 450K manifest file

**Supplementary Table ST7**. Top CpG sites (p-value <10^-5^) from the meta-analysis of epigenome-wide association studies of overall, verbal and non-verbal cognitive abilities in childhood and DNA methylation in cord blood with adjustment for maternal IQ.

|  | **CpG site** | **N** | **Beta**^a^ | **S.E.** | **P-value** ^b^ | **I^2^** ^c^ | **Chr.** | **Position** | **Gene** ^d^ |
| --- | --- | --- | --- | --- | --- | --- | --- | --- | --- |
| **Overall** | - | - | - | - | - | - | - | - | *-* |
| **Verbal** | cg16713732 | 769 | 3.85 | 0.84 | 4.32E-06 | 18.0 | 3 | 127325013 | *MCM2* |
| **Non-verbal** | cg10254690 | 1548 | -5.68 | 1.27 | 8.08E-06 | 43.3 | 10 | 126107861 | *OAT* |
|  | cg10273821 | 1579 | 2.19 | 0.49 | 8.97E-06 | 0 | 15 | 28014188 | *OCA2* |

^a^ Beta coefficient from the regression indicating the change in IQ score standard deviations per unit change in proportion methylation. Models were adjusted also for age at testing, sex, maternal age at delivery, birthweight, gestational age, maternal smoking status during pregnancy, parity, batch covariates and cell proportions

^b^ Uncorrected p-value

^c^ Heterogeneity statistics

^d^ Gene annotation from the Illumina 450K manifest file

**Supplementary Table ST8**. Top CpG sites (p-value <10^-5^) from the meta-analysis of epigenome-wide association studies of overall, verbal and non-verbal cognitive abilities in childhood and DNA methylation in cord blood with further adjustment for 10 principal components from genetic data.

|  | **CpG site** | **N** | **Beta**^a^ | **S.E.** | **P-value** ^b^ | **I^2^** ^c^ | **Chr.** | **Position** | **Gene** ^d^ |
| --- | --- | --- | --- | --- | --- | --- | --- | --- | --- |
| **Overall** | cg06760279 | 1442 | 3.51 | 0.75 | 3.05E-06 | 3.7 | 14 | 23947073 | *NGDN* |
|  | cg21899374 | 1442 | 3.16 | 0.68 | 3.84E-06 | 0 | 8 | 144915517 |  |
|  | cg23951474 | 1447 | -1.94 | 0.42 | 3.99E-06 | 20.6 | 11 | 2188061 | *TH* |
|  | cg23789148 | 1444 | 2.34 | 0.51 | 4.59E-06 | 0 | 15 | 97321146 |  |
|  | cg05264908 | 1425 | 2.40 | 0.53 | 5.80E-06 | 0 | 16 | 2049630 | *ZNF598* |
|  | cg13783152 | 1447 | 4.62 | 1.04 | 8.60E-06 | 0 | 9 | 17579026 | *SH3GL2* |
| **Verbal** | cg10334750 | 1432 | 4.04 | 0.86 | 2.54E-06 | 0 | 8 | 101348456 |  |
|  | cg05083414 | 1435 | -4.53 | 0.98 | 3.75E-06 | 51.0 | 4 | 2627039 | *FAM193A* |
|  | cg23951474 | 1452 | -1.95 | 0.42 | 4.29E-06 | 38.4 | 11 | 2188061 | *TH* |
|  | cg12162201 | 1451 | 38.17 | 8.56 | 8.21E-06 | 62.6 | 21 | 48055631 | *PRMT2* |
| **Non-verbal** | cg07805967 | 2510 | 3.55 | 0.74 | 1.42E-06 | 0 | 10 | 53459337 | *CSTF2T* |
|  | cg02366575 | 2502 | 10.25 | 2.27 | 6.17E-06 | 9.3 | 1 | 91966307 | *CDC7* |
|  | cg12813441 | 2506 | -1.61 | 0.36 | 7.88E-06 | 0 | 2 | 55239331 | *RTN4* |
|  | cg21950196 | 2512 | 2.45 | 0.55 | 8.51E-06 | 0 | 1 | 228327428 | *GUK1* |

^a^ Beta coefficient from the regression indicating the change in IQ score standard deviations per unit change in proportion methylation. Models were adjusted also for age at testing, sex, maternal age at delivery, maternal education, birthweight, gestational age, maternal smoking status during pregnancy, parity, batch covariates and cell proportions

^b^ Uncorrected p-value

^c^ Heterogeneity statistics

**Supplementary Table ST9.** Association estimates at top sites from the main model EWAS meta-analyses across all sensitivity models.

|  |  | **Main model** | | | **Paternal education** | | | **Maternal IQ** | | | **Genetic PCs** | | |
| --- | --- | --- | --- | --- | --- | --- | --- | --- | --- | --- | --- | --- | --- |
|  | **CpG site** | **Beta** | **S.E.** | **P-value** | **Beta** | **S.E.** | **P-value** | **Beta** | **S.E.** | **P-value** | **Beta** | **S.E.** | **P-value** |
| **Overall** | cg05827775 | -3.05 | 0.64 | 1.84E-06 | -2.95 | 0.66 | 7.73E-06 | -1.42 | 1.11 | 0.20 | -2.93 | 0.84 | 0.0005 |
|  | cg00213080 | -2.44 | 0.51 | 1.89E-06 | -2.48 | 0.52 | 1.48E-06 | -1.39 | 1.25 | 0.27 | -2.44 | 0.62 | 7.53E-05 |
|  | cg26599274 | 10.83 | 2.30 | 2.46E-06 | 10.58 | 2.36 | 7.17E-06 | 4.43 | 4.27 | 0.30 | 11.97 | 2.92 | 4.09E-05 |
|  | cg23789148 | 2.03 | 0.44 | 3.88E-06 | 2.12 | 0.46 | 4.60E-06 | 2.07 | 1.05 | 0.05 | 2.34 | 0.51 | 4.59E-06 |
|  | cg18622281 | 3.96 | 0.86 | 3.91E-06 | 3.90 | 0.87 | 7.76E-06 | 2.38 | 1.57 | 0.13 | 3.50 | 1.13 | 0.002 |
|  | cg00573504 | 3.71 | 0.81 | 4.98E-06 | 3.52 | 0.83 | 2.11E-05 | 2.96 | 2.48 | 0.23 | 2.93 | 0.94 | 0.002 |
|  | cg09535605 | 5.57 | 1.22 | 5.09E-06 | 5.13 | 1.23 | 2.94E-05 | 3.16 | 2.36 | 0.18 | 5.77 | 2.09 | 0.006 |
|  | cg21735491 | -9.38 | 2.09 | 6.77E-06 | -9.20 | 2.08 | 9.38E-06 | -9.13 | 7.10 | 0.20 | -10.75 | 2.85 | 0.0002 |
|  | cg18075761 | -23.68 | 5.36 | 9.84E-06 | -21.74 | 5.43 | 6.30E-05 | -11.79 | 12.80 | 0.36 | -20.66 | 6.98 | 0.003 |
| **Verbal** | cg03568675 | -3.00 | 0.59 | 4.36E-07 | -2.8 | 0.64 | 9.28E-06 | -0.81 | 1.31 | 0.54 | -2.65 | 0.70 | 0.0002 |
|  | cg12361663 | 36.38 | 7.70 | 2.30E-06 | 35.71 | 7.67 | 3.22E-06 | 35.54 | 19.19 | 0.06 | 37.28 | 11.47 | 0.001 |
|  | cg17223866 | 5.21 | 1.12 | 3.14E-06 | 3.92 | 1.19 | 0.001 | 7.38 | 2.95 | 0.01 | 5.30 | 1.27 | 3.05E-05 |
|  | cg11005998 | 2.94 | 0.64 | 3.87E-06 | 2.29 | 0.67 | 0.0006 | 2.70 | 2.43 | 0.27 | 2.41 | 0.71 | 0.0007 |
|  | cg10620273 | 22.72 | 5.00 | 5.65E-06 | 22.04 | 4.97 | 9.19E-06 | 5.49 | 10.12 | 0.59 | 10.62 | 8.13 | 0.19 |
|  | cg16047144 | -13.16 | 2.92 | 6.33E-06 | -12.01 | 2.99 | 5.88E-05 | -11.78 | 6.20 | 0.06 | -13.13 | 4.01 | 0.001 |
| **Non-verbal** | cg04783204 | 8.90 | 1.91 | 3.11E-06 | 9.76 | 1.99 | 8.97E-07 | 6.76 | 2.32 | 0.004 | 9.34 | 2.22 | 2.63E-05 |
|  | cg04229103 | 6.34 | 1.36 | 3.31E-06 | 6.58 | 1.42 | 3.74E-06 | 4.99 | 1.63 | 0.002 | 5.60 | 1.58 | 0.0004 |
|  | cg00573504 | 3.38 | 0.73 | 3.32E-06 | 3.39 | 0.76 | 7.53E-06 | 1.47 | 1.17 | 0.21 | 2.57 | 0.80 | 0.001 |
|  | cg25990848 | 2.07 | 0.45 | 3.59E-06 | 2.18 | 0.46 | 2.45E-06 | 2.92 | 1.14 | 0.01 | 2.04 | 0.49 | 3.59E-05 |
|  | cg08529049 | -4.95 | 1.08 | 4.30E-06 | -4.25 | 1.17 | 0.0003 | -5.39 | 1.45 | 0.0002 | -4.06 | 1.19 | 0.0007 |
|  | cg03332597 | 3.83 | 0.84 | 4.80E-06 | 4.30 | 0.86 | 6.49E-07 | 2.55 | 1.04 | 0.02 | 3.70 | 0.96 | 0.0001 |

**Supplementary table ST10.** Results of the random effect meta-analysis for the CpG sites with lowest p-value (<10^-5^) in the fixed effect meta-analysis of cognitive skills assessed by the Wechsler Intelligence Scale for Children in ALSPAC, PREDO and CHAMACOS at age 7-9 years.

|  | **CpG site** | **Beta** | **S.E.** | **P-value** | **I^2^** | **Gene** | **Chromosome** | **Location** |
| --- | --- | --- | --- | --- | --- | --- | --- | --- |
| **Overall** | cg12727513 | 33.32 | 6.76 | 8.25E-07 | 75.7 | *SS18L2* | 3 | 42632622 |
|  | cg00213080 | -2.79 | 0.59 | 2.33E-06 | 0 | *CYTH3* | 7 | 6204521 |
|  | cg10502231 | -3.83 | 0.83 | 3.51E-06 | 32.2 |  | 1 | 224363449 |
|  | cg04522575 | -1.31 | 0.29 | 7.46E-06 | 0 |  | 17 | 61042898 |
|  | cg19449377 | 3.81 | 0.86 | 8.45E-06 | 0 | *KRT20* | 17 | 39041199 |
|  | cg23919118 | 4.86 | 1.09 | 8.93E-06 | 25.3 | *RFX4* | 12 | 106976006 |
| **Verbal** | cg03568675 | -3.28 | 0.67 | 1.13E-06 | 40.0 | *RSPO4* | 20 | 979279 |
|  | cg17845617 | -2.49 | 0.53 | 2.20E-06 | 0 | *PIP5K1C* | 19 | 3698157 |
|  | cg11005998 | 3.31 | 0.70 | 2.56E-06 | 0 | *MIR655* | 14 | 101514642 |
|  | cg03241323 | 39.53 | 8.53 | 3.55E-06 | 0 | *ITGAV* | 2 | 187454863 |
|  | cg07838730 | 2.07 | 0.45 | 3.77E-06 | 0 | *SIGLEC15* | 18 | 43422371 |
|  | cg18167340 | 5.28 | 1.16 | 5.57E-06 | 0 | *DMAP1* | 1 | 44679636 |
|  | cg09319828 | -1.12 | 0.25 | 7.30E-06 | 11.5 | *TTC24* | 1 | 156551787 |
| **Non-verbal** | cg01323381 | -1.62 | 0.32 | 2.92E-07 | 0 | *HOXA5* | 7 | 27184264 |
|  | cg12128839 | -0.83 | 0.18 | 2.27E-06 | 0 | *HOXA5* | 7 | 27183436 |
|  | cg10502231 | -4.05 | 0.88 | 3.95E-06 | 67.8 |  | 1 | 224363449 |
|  | cg02571470 | -2.87 | 0.63 | 5.74E-06 | 68.8 | *SFXN5* | 2 | 73294845 |
|  | cg00872677 | -5.00 | 1.10 | 5.93E-06 | 50.4 | *TTLL8* | 22 | 50493126 |
|  | cg00213080 | -2.85 | 0.64 | 7.05E-06 | 0 | *CYTH3* | 7 | 6204521 |
|  | cg19274676 | 12.89 | 2.88 | 7.57E-06 | 13.7 | *KIF13A* | 6 | 17988463 |
|  | cg24587796 | 6.49 | 1.46 | 8.31E-06 | 64.1 | *RP9P* | 7 | 32979849 |

**Supplementary Table ST11.** Replication of sites found in Marioni et al. (2018): results for the top two sites across main models and results for the replicated at Bonferroni-adjusted p-value<0.05

|  | **CpG site** | **Beta**^a^ | **S.E.** | **P-value** ^b^ | **I^2^** ^c^ | **Direction of effect in Marioni et al.** |
| --- | --- | --- | --- | --- | --- | --- |
| **Overall** | cg12507869 | 0.55 | 1.22 | 0.65 | 29.0 | - |
|  | cg21450381 | 1.37 | 1.02 | 0.18 | 0 | - |
| **Verbal** | cg12507869 | 1.04 | 1.24 | 0.40 | 46.6 | - |
|  | cg21450381 | 1.45 | 1.03 | 0.16 | 0 | - |
| **Non-verbal** | cg12507869 | -0.87 | 0.92 | 0.34 | 34.5 | - |
|  | cg21450381 | NA | NA | NA | NA | - |
| **Non-verbal**  **(replicated site)** | cg17759224 | 1.17 | 0.34 | 0.00065 | 0 | + |

^a^ Beta coefficient from the regression indicating the change in IQ score standard deviations per unit change in proportion methylation. Models were adjusted also for age at testing, sex, maternal age at delivery, maternal education, birthweight, gestational age, maternal smoking status during pregnancy, parity, batch covariates and cell proportions

^b^ Uncorrected p-value

^c^ Heterogeneity statistics

NA= not available in the meta-analysis

**Supplementary Table ST12.** Replication of sites found in Karlsson-Linner et al. (2018): results for top two sites across main models and results for the replicated at Bonferroni-adjusted p-value<0.05

|  | **CpG site** | **Beta**^a^ | **S.E.** | **P-value** ^b^ | **I^2^** ^c^ | **Direction of effect in Karlsson-Linner et al. 2017** |
| --- | --- | --- | --- | --- | --- | --- |
| **Overall** | cg01940273 | -1.06 | 0.61 | 0.08 | 0 | + |
|  | cg03636183 | 0.62 | 0.44 | 0.15 | 0 | + |
|  | cg05575921 | -0.75 | 0.47 | 0.11 | 0 | + |
|  | cg05951221 | -0.07 | 0.88 | 0.94 | 0 | + |
|  | cg06126421 | NA | NA | NA | NA | + |
|  | cg12803068 | 0.02 | 0.21 | 0.94 | 47.7 | - |
|  | cg21161138 | 0.19 | 0.32 | 0.56 | 0 | + |
|  | cg21566642 | 0.65 | 0.80 | 0.42 | 0 | + |
|  | cg22132788 | 0.03 | 0.44 | 0.94 | 45.2 | - |
| **Verbal** | cg01940273 | -0.59 | 0.61 | 0.33 | 0 | + |
|  | cg03636183 | 0.52 | 0.44 | 0.24 | 0 | + |
|  | cg05575921 | -0.64 | 0.48 | 0.18 | 0 | + |
|  | cg05951221 | 0.23 | 0.89 | 0.79 | 0 | + |
|  | cg06126421 | NA | NA | NA | NA | + |
|  | cg12803068 | 0.22 | 0.21 | 0.29 | 33.8 | - |
|  | cg21161138 | -0.04 | 0.33 | 0.90 | 0 | + |
|  | cg21566642 | 0.25 | 0.81 | 0.76 | 0 | + |
|  | cg22132788 | 0.67 | 0.44 | 0.13 | 0 | - |
| **Non-verbal** | cg01940273 | -0.38 | 0.55 | 0.48 | 14.0 | + |
|  | cg03636183 | 0.70 | 0.41 | 0.09 | 0 | + |
|  | cg05575921 | -0.21 | 0.40 | 0.61 | 29.5 | + |
|  | cg05951221 | 0.74 | 0.67 | 0.27 | 2.8 | + |
|  | cg06126421 | NA | NA | NA | NA | + |
|  | cg12803068 | -0.29 | 0.19 | 0.13 | 0 | - |
|  | cg21161138 | 0.14 | 0.32 | 0.65 | 0 | + |
|  | cg21566642 | 0.85 | 0.65 | 0.19 | 0 | + |
|  | cg22132788 | NA | NA | NA | NA |  |

^a^ Beta coefficient from the regression indicating the change in IQ score standard deviations per unit change in proportion methylation. Models were adjusted also for age at testing, sex, maternal age at delivery, maternal education, birthweight, gestational age, maternal smoking status during pregnancy, parity, batch covariates and cell proportions

^b^ Uncorrected p-value

^c^ Heterogeneity statistics

NA= not available in the meta-analysis

**Supplementary Table S13**. Differentially methylated region for non-verbal cognitive skills from the meta-analysis adjusted for maternal IQ at Bonferroni-corrected p<0.05

| **DMR** | | | | **Maternal IQ** | | | | |
| --- | --- | --- | --- | --- | --- | --- | --- | --- |
| **CpG site** | **Chr.** | **Position** | **Gene** ^d^ | **N** | **Beta**^a^ | **S.E.** | **P-value** ^b^ | **I^2^** ^c^ |
| cg03395511 | chr6 | 291903 | *DUSP22* | 1604 | 0.26 | 0.17 | 0.121 | 0 |
| cg07332563 | chr6 | 291687 | *DUSP22* | 1862 | 0.34 | 0.13 | 0.009 | 0 |
| cg15383120 | chr6 | 291909 | *DUSP22* | 1804 | 0.27 | 0.12 | 0.029 | 0 |
| cg18110333 | chr6 | 292329 | *DUSP22* | 1862 | 0.26 | 0.10 | 0.012 | 5.3 |
| cg21548813 | chr6 | 291882 | *DUSP22* | 1804 | 0.26 | 0.13 | 0.041 | 0 |

^a^ Beta coefficient from the regression indicating the change in IQ score standard deviations per unit change in proportion methylation.

Covariates in the maternal IQ model were: age at testing, sex, maternal age at delivery, birthweight, gestational age, maternal smoking status during pregnancy, parity, batch covariates, cell proportions and maternal IQ.

^b^ Uncorrected p-value

^c^ Heterogeneity statistics

**Supplementary Table S14**. Differentially methylated region for non-verbal cognitive skills from the meta-analysis adjusted for maternal IQ at Bonferroni-corrected p<0.05, across other models

|  | **Main model** | | | | | **Paternal education** | | | | | **Genetic PCs** | | | | |
| --- | --- | --- | --- | --- | --- | --- | --- | --- | --- | --- | --- | --- | --- | --- | --- |
| **CpG site** | **N** | **Beta**^a^ | **S.E.** | **P-value** ^b^ | **I^2^** ^c^ | **N** | **Beta**^a^ | **S.E.** | **P-value** ^b^ | **I^2^** ^c^ | **N** | **Beta**^a^ | **S.E.** | **P-value** ^b^ | **I^2^** ^c^ |
| cg03395511 | 2976 | 0.003 | 0.13 | 0.98 | 13 | 2712 | 0.08 | 0.13 | 0.53 | 9.7 | 1364 | 0.02 | 0.16 | 0.91 | 0 |
| cg07332563 | 3300 | 0.21 | 0.11 | 0.05 | 0 | 3013 | 0.26 | 0.11 | 0.03 | 0 | 1454 | 0.26 | 0.13 | 0.05 | 18.1 |
| cg15383120 | 3212 | 0.11 | 0.10 | 0.27 | 29.9 | 2925 | 0.16 | 0.10 | 0.13 | 25.8 | 1407 | 0.10 | 0.12 | 0.41 | 34.6 |
| cg18110333 | - | - | - | - | - | 2939 | 0.15 | 0.09 | 0.09 | 30.3 | 1411 | 0.11 | 0.10 | 0.26 | 48.2 |
| cg21548813 | 3210 | 0.10 | 0.11 | 0.37 | 20.9 | 2923 | 0.14 | 0.11 | 0.23 | 21.1 | 1401 | 0.09 | 0.13 | 0.49 | 0 |

^a^ Beta coefficient from the regression indicating the change in IQ score standard deviations per unit change in proportion methylation.

Covariates in the models were:

Main model: age at testing, sex, maternal age at delivery, maternal education, birthweight, gestational age, maternal smoking status during pregnancy, parity, batch covariates, cell proportions.

Paternal education model: age at testing, sex, maternal age at delivery, maternal education, birthweight, gestational age, maternal smoking status during pregnancy, parity, batch covariates, cell proportions, paternal education.

Genetic PCs model: age at testing, sex, maternal age at delivery, birthweight, gestational age, maternal smoking status during pregnancy, parity, batch covariates, cell proportions and genetic PCs.

^b^ Uncorrected p-value

^c^ Heterogeneity statistics

**Supplementary Table S15.** Correlation between DNA methylation and gene expression at the *DUSP22* differentially methylated region (https://genenetwork.nl/biosqtlbrowser/).

| **CpG** | **P-value** | **CpG**  **Chr.** | **CpG Chr.**  **Position** | **Probe** | **Probe**  **Chr.** | **Probe Chr.**  **position** | **Z-score** |
| --- | --- | --- | --- | --- | --- | --- | --- |
| cg03395511 | 2.63E-154 | 6 | 291855 | ENSG00000112679 | 6 | 292097 | 26.46 |
| cg07332563 | 4.11E-163 | 6 | 291639 | ENSG00000112679 | 6 | 292097 | 27.22 |
| cg15383120 | 4.4E-160 | 6 | 291861 | ENSG00000112679 | 6 | 292097 | 26.96 |
| cg18110333 | 3.83E-155 | 6 | 292329 | ENSG00000112679 | 6 | 292097 | 26.53 |
| cg21548813 | 2.44E-157 | 6 | 291834 | ENSG00000112679 | 6 | 292097 | 26.72 |
